# Is There a Fly in My Soup? To What Extent Do Metabarcoding and Individual Barcoding Tell the Same Story?

**DOI:** 10.1111/1755-0998.70195

**Published:** 2026-09-11

**Authors:** Brendan Furneaux, Tomas Roslin, Bess Hardwick, Deirdre Kerdraon, Hannu Autto, Gaia Banelyte, Jeremy R. deWaard, Stephanie L. deWaard, Arielle Farrell, Oula Kalttopää, Erik Kristensen, Hanna M. K. Rogers, Jayme E. Sones, Evgeny V. Zakharov, Otso Ovaskainen

**Affiliations:** ^1^ Department of Biological and Environmental Science University of Jyväskylä Jyväskylä Finland; ^2^ Department of Ecology Swedish University of Agricultural Sciences Uppsala Sweden; ^3^ Organismal and Evolutionary Biology Research Programme, Faculty of Biological and Environmental Sciences University of Helsinki Helsinki Finland; ^4^ Kilpisjärvi Biological Station University of Helsinki Helsinki Finland; ^5^ Centre for Biodiversity Genomics University of Guelph Guelph Ontario Canada; ^6^ Unit for Field‐Based Forest Research Swedish University of Agricultural Sciences Umeå Sweden; ^7^ Department of Integrative Biology, College of Biological Sciences University of Guelph Guelph Ontario Canada

## Abstract

Metabarcoding has become the method of choice for characterizing complex arthropod communities. The extent to which metabarcoded bulk samples will recover the same community composition as individual sequencing of all individuals in the sample remains poorly quantified. Biases such as unequal extraction of DNA from different taxa, primer mismatches and non‐random PCR may cause the selective drop‐out of species from metabarcoding data. At the same time, DNA metabarcoding may reveal arthropod taxa present not as individuals, but as DNA residues on the surface or in the gut of insects. To quantify the consistency in sample contents established by different means, we metabarcoded 45 bulk insect samples, then extracted all arthropods and sequenced them individually. Metabarcoding targeted 418 bp at the 3′ end of the Folmer barcoding region, while individual barcodes captured the entire 658 bp Folmer region. The metabarcoding workflow, including PCR amplification, sequencing and bioinformatics, was performed in three replicates from three separate lysate aliquots per sample. For the main analyses, sequences were assigned to Barcode Index Numbers (BINs) as identical taxonomic categories across data types, thereby allowing the detection of even rare but biologically true taxa. Since such reference‐based validation will be unavailable to any researcher dealing with metabarcoding data alone, we validated our key findings through an alternative workflow, i.e., de novo clustering of sequences. We found that metabarcoding is replicable, as different replicates of the same sample recover similar species richness and composition. Individual barcoding and metabarcoding provide similar impressions of relative differences in community structure: species‐rich vs. species‐poor samples rank similarly among data types (Spearman's *⍴* = 0.88–0.99) as do differences in relative dissimilarity between sample pairs (Spearman's *⍴* = 0.55–0.90). Dissimilarity between data types varies with BIN richness in the sample, but this relationship reflects nestedness rather than turnover: metabarcoding recovers the same set of core species as individual barcoding but adds hundreds of species on top. Any BIN recovered as an individual occurred with high probability in the metabarcoding data, and any BIN found in high read abundances by metabarcoding was likely found as an individual (*p* > 0.8). In terms of abundances, the number of individual insects per BIN was well predicted by the number of metabarcoding reads (*R* ^2^ > 0.68 for a model including taxonomy as a random effect). Our analysis suggests that metabarcoding data will be informative of the sample contents in terms of arthropod species richness, composition and taxon‐specific abundances. Taxa recovered in low copy numbers in metabarcoding sequence data will likely represent DNA left as residues from past biotic interactions. Barring sequencing errors, both types of data yield biologically relevant insights into the taxa present in the source community.

## 1 Introduction

Metabarcoding of arthropod samples is rapidly becoming the go‐to method for biodiversity discovery (Cristescu 2014; Souto‐Vilarós et al. 2024; Yu et al. 2012), community comparisons (Barsoum et al. 2019; Taberlet et al. 2012), ecological monitoring (Granqvist et al. 2025; van Klink et al. 2022, 2024) and testing of topical theories in community ecology (Abrego et al. 2021; Andújar et al. 2022). Indeed, metabarcoding is arguably the only current method allowing us to thoroughly characterize large and complex arthropod samples at a relevant scale, such as the ca. 20,000 Malaise trap samples collected by the LIFEPLAN project (Hardwick et al. 2024; Ovaskainen et al. 2025). Yet, all new types of data come with both benefits and caveats, and metabarcoding data are no exception (Hartig et al. 2023). A concern repeatedly raised is the extent to which metabarcoding will suffice to reveal *all* taxa actually present in the sample, i.e., the ‘true sample content’—typically taken to be the species present as recognizable individuals (Basset et al. 2022; Elbrecht et al. 2017; Yu et al. 2012). When metabarcoding fails to detect species which might, in principle, have been detectable by a (very large) set of skilled taxonomists or the (expensive and tedious) individual barcoding of all individuals present in the sample (as done in Global Malaise Trapping Project; Geiger et al. 2016; Seymour et al. 2024), then the method is often dismissed as incomplete or biased. When samples reveal taxa not present as individuals, they are often taken to represent contamination, misidentifications or false positives (Iwaszkiewicz‐Eggebrecht et al. 2023; Ji et al. 2020). In terms of accurately recovering taxon‐specific abundances beyond presence/absence, metabarcoding has been labelled as being semi‐quantitative at best (Lamb et al. 2019), although multiple initiatives are trying to improve on the status quo (Shelton et al. 2023; Iwaszkiewicz‐Eggebrecht et al. 2026).

There are many reasons why species present in the sample as individuals may (and typically will) be lacking from metabarcoding data, and why read numbers may not accurately reflect abundances (Lamb et al. 2019). Well‐known and amply studied reasons include unequal extraction of DNA from taxa of different structure (Braukmann et al. 2019; Marquina et al. 2022), primer mismatches and PCR biases (Elbrecht et al. 2019; Piñol et al. 2019), and the swamping of signal from rare species by more abundant ones in sequence yields of realistic size (Elbrecht and Leese 2015). Each of these factors may result in the systematic underrepresentation of selected taxa, thereby causing a bias in the data and downstream analyses. The basic chemistry of PCR and sequencing reactions is also associated with stochasticity, generating random noise (Leray and Knowlton 2017; Shelton et al. 2023). Given the large number of DNA templates present in a complex sample, some templates may get amplified by chance alone, whereas others fail to do so (Piñol et al. 2019). As a result, samples processed in replicates typically show some differences in the exact species pool recovered (Van Den Bulcke et al. 2021; Zinger et al. 2019), with individual species ‘dropping out’ from the sequence yield of individual replicates.

The challenge of ‘false negatives’ (i.e., species present in the sample but not detected in the metabarcoding data) has been studied *in silico* (Ficetola et al. 2015, 2016; Lahoz‐Monfort et al. 2016), in vitro (Piñol et al. 2015), and empirically, through the creation of so‐called ‘mock communities’ of known contents (Elbrecht et al. 2019; Elbrecht and Leese 2015; Martoni et al. 2022; Yu et al. 2012). Such communities are typically assembled from a handful to some tens of species (Blanckenhorn et al. 2016; Iwaszkiewicz‐Eggebrecht et al. 2023; Piñol et al. 2019; but for more ambitious approaches, see Creedy et al. 2019; Elbrecht et al. 2019). A common finding is that detection rates are never perfect. Some taxa (such as ants; Basset et al. 2022; Iwaszkiewicz‐Eggebrecht et al. 2023) appear to be particularly hard to detect—and poor detectability can sometimes be attributed to simple correlates, including a hard and sclerotized surface (Iwaszkiewicz‐Eggebrecht et al. 2023; Marquina et al. 2022).

Yet, there are also perfectly good reasons why species *not* present in the sample as individuals will appear in the sequence yield. In particular, a large number of studies have shown how individual arthropods will accumulate DNA traces of past interactions (Borsato et al. 2025; Wirta et al. 2022), from DNA in gut contents (Eitzinger et al. 2019; Kocher et al. 2017; Wirta et al. 2015) to DNA from past hosts or current parasitoids (Wirta et al. 2014). Critically scanning for ‘false positives’ (i.e., species appearing in the sample even when no one put them there) will thus be an unrewarding task—unless the community was generated from laboratory‐reared individuals fed on DNA‐free food.

Given the challenges involved both in establishing biologically‐relevant mock communities, and in defining the ground‐truth based on *either* metabarcoding data *or* individually‐sequenced specimens, we suggest a more constructive approach: to quantify the factors affecting the probability by which a taxon detectable as an individual in the sample is also detected in the metabarcoding data, and—*mutatis mutandis*—the probability with which a taxon detected in the metabarcoding data will also be present as an individual. In this paper, we draw on a set of 45 samples exhaustively characterized by both metabarcoding and sequencing of individuals to achieve this. More specifically, we ask:

1. Is metabarcoding in itself reproducible: do different replicates of the same samples recover the same species richness and the same species composition from a sample?
2. Do individual barcoding and metabarcoding provide the same impression of community structure:
  2.a Do estimates of species richness concur among methods?
  2.b Do estimates of compositional differences between samples concur among methods?
  2.c Does the concurrence between data types depend on the bioinformatics pipeline applied, or on the species richness in the sample?
3. Can we predict the probability of species occurrence in one type of data by its abundance in the other type (*sensu* individuals for individual barcodes and read counts for metabarcoding data)?
4. Can we predict the abundance of individuals in the original sample from read counts observed in metabarcoding data?

## 2 Materials and Methods

### 2.1 Field Sampling and Data Generation

Forty‐five insect samples were collected by Malaise traps across 19 sites of which 18 were located in Sweden, and the last near the border in Finland. All data and the underlying methods were released by Orsholm et al. (2026). In brief, we first metabarcoded the samples following the protocol outlined by deWaard et al. (2024). Prior to DNA extraction, ethanol was drained from the samples and the total wet biomass of each sample was measured. To assess the reproducibility of metabarcoding, we split the DNA extract from each bulk sample into three replicates and processed these independently through PCR, library preparation, sequencing, and bioinformatics. The forty‐five focal samples were metabarcoded in mixed DNA extraction and sequencing batches along with 675 other Malaise trap samples from the LIFEPLAN project (Hardwick et al. 2024) at the Centre for Biodiversity Genomics (CBG) in Guelph, Canada in the two lanes of a single Illumina NovaSeq 6000 run. To establish the level of cross‐contamination, each 96‐well PCR plate included thirty biological samples (ninety PCR replicates) as well as three positive controls and three template‐free negative controls for both PCR and sequencing. The positive controls contained a synthetic mock community of non‐biological sequences based on SynMock (Palmer et al. 2018), modified to include the target BF3‐BR2 primer sites. The positive control sequences were filtered out by both bioinformatics pipelines (see below) and thus results for the positive and negative controls were treated together. This approach was based on the rationale that once any sequence representative of the synthetic mock community has been removed, any remaining sequences will serve as indicators of contamination. In analyses where the three replicates of each sample were combined (see below), we treated the three positive controls and three negative controls as replicates and combined them in the same way.

After DNA extraction for metabarcoding, we sorted all detectable individual arthropods from the forty‐five focal samples and exhaustively DNA barcoded them following Ivanova et al. (2006) for DNA extraction and Hebert et al. (2018) for sequencing. Individual arthropods from each sample were sorted roughly by size, with all specimens (intact, damaged or broken) included. Specimens smaller than 5 mm were arrayed directly into an 8 × 12 (96‐well) microplate. Specimens larger than 5 mm were each placed into a 5 mL tube and arrayed into batches of 96 to align with the 8 × 12 (96‐well) microplate format. Each 96‐well array was given a unique identifier (BIOUG#####) and each specimen was given a unique sample ID based on the array and its location within it (e.g., BIOUG12345‐A01). All taxonomy was assigned to ‘Arthropoda’ by default. Specimen records, including all collection information, were generated for each individual and uploaded to the CIMS (Collections Information Management System) in a batch. These records were then uploaded to the online platform, Barcode of Life Data systems (BOLD; Ratnasingham and Hebert 2007), along with high‐resolution images of each specimen. Specimens in microplates were photographed with the Keyence VHX System (Steinke, Ratnasingham, et al. 2024), whereas larger individuals were pinned and then individual images were captured using the Automatic Imaging Rig (Steinke, McKeown, et al. 2024). Images were uploaded to their relevant Specimen Records on BOLD, where they are run through an AI Order‐level ID classification to update their taxonomy accordingly. For the full set of images, see Orsholm et al. (2026). All resulting microplates and tube arrays were submitted to the lab for whole voucher analysis (no subsampling of specimens).

The individual specimens subsequently underwent Multigene—2021_SEQUEL 96 GFP DNA Extraction. Here, automated, magnetic bead‐based DNA extraction was performed in 96‐well format. To maximize DNA yield, tissue lysis was done overnight at 56°C before DNA purification. After lysis was complete, all vouchers were returned to the CBG's specimen archive for permanent storage. Aliquots of DNA extracts from four 96‐well microplates were consolidated into a 384‐well PCR plate for subsequent PCR amplification to reduce reagent volumes. All DNA plates are archived in CBG's −80°C freezer archive.

For sequencing, Unique Molecular Identifiers (UMIs), present in each PCR well, were incorporated into the amplicons to be used for mapping sequence reads to vouchered specimens. PCR amplification targeted the barcode region of the cytochrome c oxidase subunit I (COI) gene. Twenty‐four 384‐well PCR plates, each containing unique UMI combinations, were pooled (hereafter referred to as a ‘SMRT cell’) and a subsample taken for Single‐Molecule Real‐Time (SMRT) sequencing. The SMRT cells were prepared for sequencing by ligating uniquely tagged SMRTbell adapters to each pool. To create the final sequencing library, the SMRT cells were pooled before annealing the sequencing primer and polymerase. The resulting library was loaded into a single SMRT cell chip to be run on the Sequel IIe Sequencing Platform.

### 2.2 Bioinformatics

**FIGURE 1:**
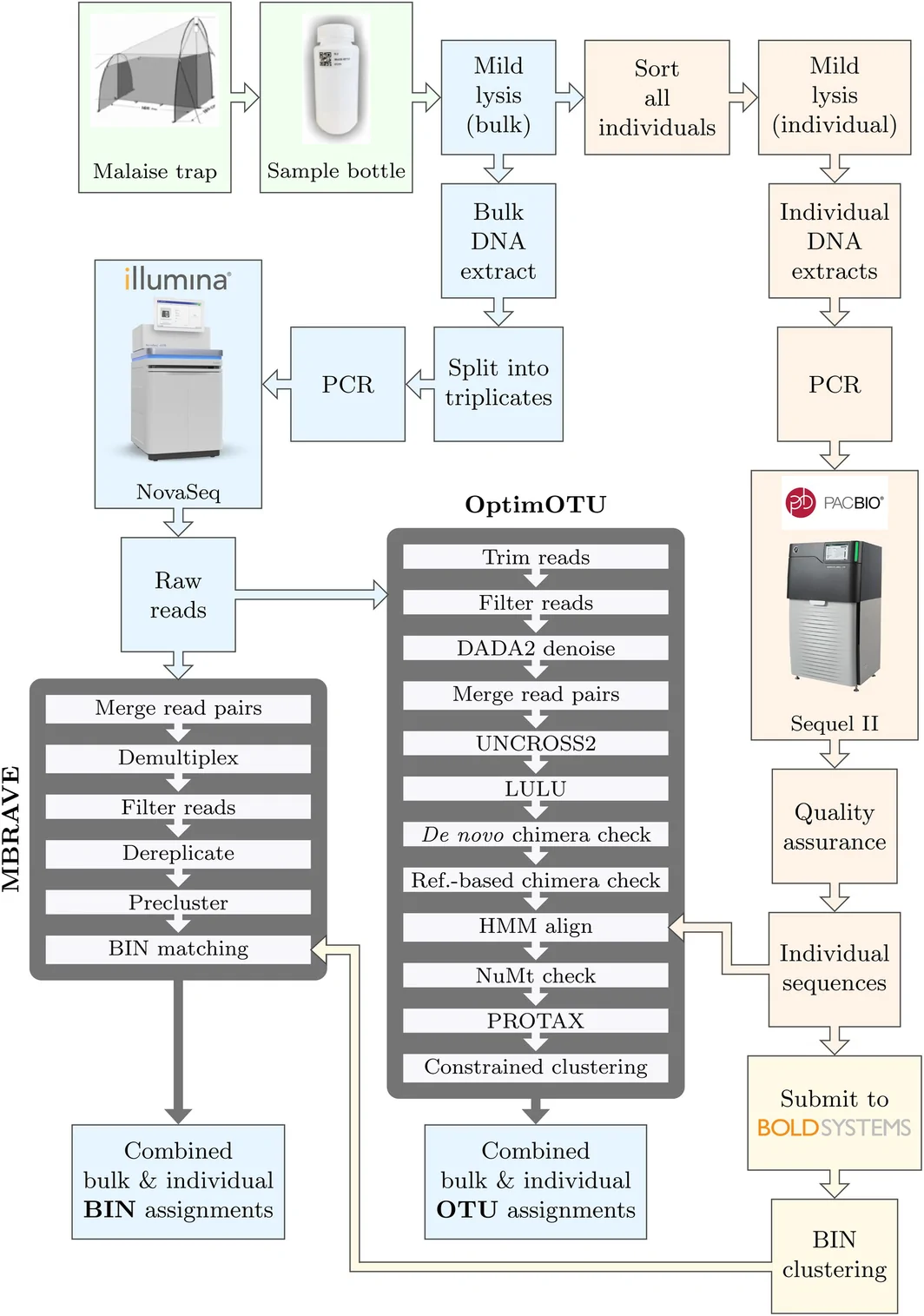
Workflow adopted. This workflow diagram summarizes the full experimental design and analytical pipeline, from sample collection through lysate preparation, replicate metabarcoding, individual barcoding, BIN assignment, and subsequent statistical analyses. Results of the reference‐based mBRAVE analysis (left) are reported in the main text, whereas results of *de novo* clustering (middle, labelled OptimOTU) are reported in Supplement S1.

Incomplete databases are some of the major limitations of metabarcoding (Keck et al. 2023). As a key challenge to real‐life metabarcoding exercises, any researcher will thus be faced with the choice of whether to trust novel sequences not observed in the reference library as traces of true taxa or as spurious sequence variants generated by the methods employed. To address this issue, we based our main analyses on a reference‐based approach. Thus, we added any Barcode Index Number (BIN) represented by real individuals in the sample to the reference set to which metabarcoding reads are compared. This will avoid the case where real BINs included in the sample (as individuals) are filtered out for being rare. Our approach yields an important advantage for addressing the questions at the core of our analyses, since it allows us to evaluate the probability that truly rare sequences still represent species present in the sample. Nonetheless, this scenario will depart from any analysis based on metabarcoding data alone, since here there is no separate data on what taxa to expect. Thus, in most analyses, the researcher will have to adopt a series of steps for inferring what sequence variants are real and which are not. Here, rare sequence variants offer a particular challenge, since occasional sequencing errors are more likely to generate rare sequence variants rather than dominant ones. As a result, most bioinformatic pipelines treat singletons and low‐abundance variants with caution and often exclude them by default (Hakimzadeh et al. 2023). The resulting difference in data raises the concern of whether conclusions from the approach outlined above will also apply to the case where all we have are metabarcoding data. To address this question, we added a comparative perspective using a *de novo* pipeline. The two workflows adopted are visually summarized in Figure 1, with figures presenting results of the reference‐based approach reported in the main text and figures presenting the *de novo* clustering reported in the Supporting Information. Overall, we note that the two workflows yield qualitatively consistent results.

#### 2.2.1 Individual‐Level Sequences

Following sequencing, a high‐quality consensus sequence/read was created during CCS (Circular Consensus Sequence) analysis. Demultiplexing mapped each sequence/read to its SMRT cell (using the SMRTbell adapter) and source specimen (using the UMIs that were added during PCR). Sequences were assigned to each specimen and underwent internal validation before being uploaded to BOLD (Ratnasingham and Hebert 2007). Barcode sequences from the individual specimens were added to BOLD as dataset DS‐LPEPA22. Once stored in BOLD, they were incorporated into the next regularly scheduled run of the BOLD Refined Single Linkage (RESL) algorithm, which assigned them, along with the rest of the sequences in BOLD, to existing barcode identification numbers (BINs; Ratnasingham and Hebert 2013), or used them to delineate new BINs if they did not match any existing BIN. Thus, the procedure and quality checks adopted in validating BINs and generating new ones exactly matched the criteria used in the rest of BOLD.

##### 2.2.1.1 Reference‐Based Approach (mBRAVE)

In the reference‐based approach, the metabarcoding sequences were processed using the mBRAVE on‐line workbench (Ratnasingham 2019; details from personal communication with S. Ratnasingham), which runs a pipeline written in Python 2.7 on a computing cluster. After merging read pairs and demultiplexing, reads with lengths < 20 or longer than 50,000 bp were removed, and remaining reads were divided into chunks of approximately 500,000 reads, which were processed in parallel for all further steps. Reads were then filtered with a more stringent length filter, removing all reads of < 446 or more than 1000 bp. The first and last 23 bp, corresponding to the expected primer locations, were then trimmed from each read, and the trimmed reads filtered by quality scores. Reads were removed if their average quality score was < 20, if more than 6% of nucleotides had a score < 20, or if more than 1% of nucleotides had a quality score < 10. After quality filtering, the remaining sequences were dereplicated, i.e., clustered at 100% identity. The mBRAVE pipeline has an optional preclustering stage after dereplication, but we did not enable it for this analysis. Dereplicated unique sequences were then matched to a series of reference database using USEARCH v8.1.186 (Edgar 2010), with a minimum match identity of 98%. Matches were selected from each database in order, with sequences matched earlier databases dropped from queries for later databases in the list. We used DS‐LPEPA22, the individual barcodes produced from the forty‐five focal samples, as the highest priority database, followed by the standard mBRAVE COI system databases: SYS‐MBRAVEC containing common contaminants, SYS‐CRLBACTERIA containing bacteria, SYS‐CRLNONARTHINVERT containing animals except phyla Arthropoda and Chordata, SYS‐CRLNONINSECTARTH containing arthropods except class Insecta, SYS‐CRLDIPTERA containing order Diptera, SYS‐CRLNONDIPINSECTA containing insects except class Diptera, SYS‐CRLCHORDATA containing phylum Chordata except class Aves and humans, SYS‐CRLAVES containing class Aves, SYS‐CRLPROTISTA containing protists, and finally SYS‐HUMC containing human sequences as a contamination check. The full mBRAVE pipeline also includes further stages to cluster remaining sequences which could not be matched to any of the reference databases at the required identity threshold, but our analysis only considers the sequences which were mapped to BINs. We discarded sequences from the metabarcoding dataset which were not mapped to BINs assigned to phylum Arthropoda. Because BINs are at present only generated for sequences derived from individual specimens, this means that all sequences retained for analysis can in principle be linked to a physical specimen, whether from the present study or another. Thus, we adopted BINs as our operational species definition. For the Nordic fauna here targeted, BINs have been shown to offer a close match to Linnaean species (Roslin et al. 2022 and references therein). As a result of these choices, any taxa appearing in the metabarcoding and individually sequenced data were defined by the same criteria and directly matchable between the two data types. At the same time, the species richness recorded in the metabarcoding is likely to be an underestimate, because we included only species that could be mapped to existing BINs rather than e.g., clustering the raw sequences.

#### 2.2.2 De Novo Clustering (OptimOTU)

Bioinformatics for the *de novo* clustering approach was performed using the OptimOTU pipeline version 7 (Furneaux et al. 2025). Because it does not rely on direct mapping to existing reference clusters (BINs), the OptimOTU pipeline includes a number of steps aimed at removing sequencing artefacts, including primary denoising using DADA2 (Callahan et al. 2016), secondary denoising using LULU (Frøslev et al. 2017), cross‐contamination removal using UNCROSS2 (Edgar 2018), chimera detection using the DADA2 consensus *de novo* method and reference‐based UCHIME (Edgar et al. 2011), and off‐target/pseudogene detection using HMM alignment (Eddy 2011; Porter and Hajibabaei 2021). The final clustering stage was performed entirely *de novo* by single‐linkage clustering with a 98% identity threshold, with the sequences produced from individual barcoding included in the clustering process, so that they can be linked to the same operational taxonomic units (OTUs) as the metabarcoding sequences. For full details on the *de novo* clustering workflow, we refer the reader to Supplement S1.

### 2.3 Statistical Analyses

All the below analyses were repeated for both the reference‐based data and data generated by *de novo* clustering of sequences. We thus use the general term OTU to refer both to the BOLD BIN‐mappings produced by mBRAVE and to the *de novo* clusters generated by OptimOTU; however, when referring specifically to the mBRAVE data, we use the more specific term BIN. All statistical analyses were performed in R 4.4.3 (R Core Team 2025).

To first evaluate the consistency between replicates and sequencing methods, we used as metrics species richness, and pairwise dissimilarity in species composition by Sørensen's and Simpson's indices. Sørensen's dissimilarity is defined by (*b* + *c*)/(2*a* + *b* + *c*), where *a* is the number of OTUs detected in both replicates, and *b* and *c* are the numbers of OTUs detected in only the first or only the second replicate, respectively (Sørensen 1948). Thus, the dissimilarity is the number of OTUs found in only one of the two samples, divided by the sum of the total number of OTUs found in each sample. This index reaches a value of zero when all OTUs are identical between samples and one when all OTUs are different. Sørensen's index is known to be sensitive to differences in OTU richness—since any species detected uniquely by one method (*b* or *c* in the formula above) tend to drive the value of the index towards its maximum value of 1. As the metabarcoding data tended to detect more species than individually sequenced samples, we wanted to assess whether metabarcoding resolved *the same plus more* OTUs than those detected by individual barcoding, or *totally different* OTUs than detected by individual barcoding. The first phenomenon is referred to as nestedness (Baselga 2010, 2012), since it is best illustrated by the case where all OTUs in the more OTU‐poor sample are also included in the more OTU‐rich sample (i.e., the species pools are perfectly nested). The second case is referred to as turnover, since it focuses on differences (i.e., turnover) in species identity. To resolve between these scenarios, we drew on another measure of community composition, Simpson's (1943) distance. This metric was originally designed to be insensitive to differences in species richness. It is defined as min(*b*, *c*)/(*a* + min(*b*, *c*)), with *a*, *b*, and *c* as defined above—i.e., the fraction of OTUs in the replicate with fewer OTUs which are unique to that replicate. Thus, it reaches a value of zero when the OTUs in the less OTU‐rich replicate are a subset of the OTUs in the more OTU‐rich replicate and a value of one when the two replicates have completely different OTUs. In the case where the two replicates have the same total number of OTUs, Sørensen's and Simpson's dissimilarities are equal (Baselga 2010). Drawing on this rationale, we repeated the analyses described below using Simpson's distance index.

To assess the replicability of metabarcoding in terms of species richness, we fitted a linear model of OTU richness explained by the sample (factor with 45 levels). The model was fitted in R using function lm, and we measured replicability by the *R* ^2^ of this model. To assess the replicability of metabarcoding in terms of species composition, we compared the Sørensen and Simpson dissimilarities between different replicates deriving from each sample to the dissimilarity between these and replicates deriving from other samples.

To assess the comparability of metabarcoding and individual barcoding in terms of species richness, we fitted a linear model of OTU richness detected by metabarcoding as a function of the OTU richness recovered by individual barcoding. Here, the intercept (if positive) is an estimate of how many arthropod OTUs were detected by metabarcoding in samples that had a very low (zero) number of OTUs detected as individuals. The slope reveals how many new OTUs we detect by metabarcoding for every new OTU detectable by sequencing of individuals. If each OTU were detected by both methods, then we would expect an intercept of zero and a slope of one. The *R* ^2^ of the linear model indicates how well we may predict species richness detected by one method based on the species richness detected by the other method. The model was fitted in R using function lm. To assess the comparability of metabarcoding and individual barcoding in terms of species composition, we computed the Sørensen and Simpson dissimilarities between the individual‐level results from a sample and the three metabarcoding replicates from the same sample. We then used a Mantel test to compare the observed Pearson's product–moment correlation coefficient, *r*, between the observed, pairwise dissimilarities to the expected distribution of *r* when the data were reshuffled among sample pairs within the other data type. This analysis was implemented using the vegan package (Oksanen et al. 2025) with 999 permutations. As we expected that low OTU richness would lead to larger nestedness, we tested whether the Sørensen and Simpson dissimilarities between the two sampled types covaried with the OTU richness resolved by barcoding of individuals or by metabarcoding. Since the two methods detected slightly different numbers of OTUs, we evaluated patterns against both OTU richness resolved by individual barcoding and OTU richness resolved by metabarcoding.

Next, we assessed how the abundance of a OTU in one type of data was reflected in its detection probability in the other type of data—e.g., whether taxa found to be rare in one type of data are likely to be missed by the other type of data. For each data type (metabarcoding or individual barcoding), we fitted a logistic regression model of the presence/absence of a OTU in data generated by one method as a function of the log(number of individuals or reads) by the other method. Each OTU in each sample was used as an individual data point. Here, the intercept will reflect the basic probability that a OTU present in a sample of one data type will be detected by the other. When metabarcoding data are used as the response, the slope will reflect how quickly this probability increases with logarithmically more individuals in the sample. When individual barcoding data are used as the response, the slope will reflect how quickly this probability increases with logarithmically more reads in the sequence yield. As a second predictor, we included log(sequencing depth) as a covariate. To see why, consider a sample with low sequencing depth (i.e., few reads in total). For such a sample, all reads—regardless of whether the OTU is present as an (or several) individual(s) or not—will be rarer than in a sample of deep sequencing depth, and the probability of a OTU yielding zero reads will thus be higher, leading to a lower detection chance in metabarcoding. Thus the expected sign of this predictor is positive when presence in metabarcoding reads is used as the response. Conversely, each read which is found for an OTU in a low‐depth sample represents a larger fraction of the total than in a high‐depth sample, and so it may be expected to represent a larger number of individuals. Thus the sign of the sequencing depth predictor is expected to be negative when the response variable is presence as an individual. To assess whether different taxonomic groups differ in detectability between the two methods (for instance due to differences in size or extractability), we fit a second model which added a random effect based on the taxonomy of the OTU at the order, family, and genus level. The taxonomic random effect was nested at the order, family, and genus level, thus allowing the detectability of different orders to vary around the overall mean detectability; within each order, different families to vary around the order‐level mean; and within each family, different genera to vary around the family‐level mean. BINs in the mBRAVE analysis which were not identified to family‐ or genus‐level were treated as having no random effect at the unidentified levels, instead using the mean detectability from the order‐ or family‐level, respectively. OptimOTU produces ‘pseudotaxon’ clusters at all ranks for all OTUs, which were used as proxies for unidentified taxonomic groups for the purpose of model fitting. We tested the predictive power of the model with taxonomic random effects using sample‐wise leave‐one‐out cross validation. That is, for each of the forty‐five samples, we fit the model to data from only the other forty‐four samples, and then predicted the presence of each OTU in the held‐out sample. Finally, to test for additional sample‐specific effects which are not captured by the sequencing depth alone, we fit a third model, which in addition to the taxonomic random effect also included a sample‐specific random effect. All three models were fit using the glmmTMB package version 1.1.14 (McGillycuddy et al. 2025) with binomial family and logit link function. To estimate the explanatory power of each model, we used Tjur's *R* ^2^, which is the difference between the mean probability of presence assigned by the model to real presences and the mean probability or presence assigned to real absences. Like the typical *R* ^2^, Tjur's *R* ^2^ has a value of 1 when the model completely captures all variation in the data (i.e., assigns probability 1 to all presences, and probability 0 to all absences) and a value of 0 for an intercept‐only model (i.e., assigns the same probability in all cases).

As our last step, we asked whether the abundance of individuals of each OTU in the original sample could be predicted from metabarcoding reads. To avoid redundancy with analyses of presence/absence (above), we used a hurdle approach, predicting individual abundance conditional on presence in both bulk (meta‐) and individual barcoding. We fit three models using the same R functions and the same sets of predictors as for the presence/absence models above, but with a zero‐truncated Poisson family and log link in order to model positive‐definite count data. As an estimate of the explanatory power of each model, we used *R* ^2^, and as an estimate of predictive power, we used *R* ^2^ based on sample‐wise leave‐one‐out cross validation.

## 3 Results

The full metabarcoding run resulted in a total of 975 million raw reads, of which 44 million were demultiplexed to one of the study samples or associated controls (Table 1). After processing through the mBRAVE pipeline, 29.7 million reads from the study samples were successfully mapped to BINs, with a mean of 213,000 reads per sample replicate (SD 59,000, range 89,000–361,000). Control sample replicates had a mean of 221 reads per sample replicate mapped to BINs (SD 320, range 17–15,900). When resolved by metabarcoding, individual sample replicates contained between 84 and 697 BINs (mean 250 ± SD 144) and control sample replicates contained between 14 and 48 BINs (mean 25 ± SD 6). The total number of BINs recovered from the metabarcoding data was 6417, of which 3194 (50%) were annotated at species level and 3223 (50%) were not.

**TABLE 1:** Summary statistics on the overall data.

Sample set	Total	Study samples	Positive controls	Negative controls
*n*	768 × 3	45 × 3	12 × 3	12 × 3
Raw	975,163,733	—	—	—
Demultiplexed	665,435,366	43,654,785	404,224	8985
Filtered	482,682,811	32,012,728	282,991	5397
Mapped	415,966,240	29,793,457	8114	4574
Arthropod	359,452,442	29,665,223	4513	4522

*Note:* Shown are the numbers of samples of different type and number of reads passing each step in the bioinformatic pipeline used for reference‐based analyses. The positive control and negative control columns refer only to control samples belonging to a 96‐well PCR plate which also included at least one of the study samples.

In the OptimOTU pipeline, more reads were filtered out, with reads primarily removed by the early application of a stringent filter on quality score (Figure 1; Table S1). This yielded a total of 14.8 million reads from the study samples, with a mean of 109,000 reads per sample replicate (SD 29,000, range 47,500–176,000). A mean of 5.9 reads was recovered per control replicate (SD 14.5, range 0–64). The lower read counts also corresponded to lower OTU richness, with 24–328 OTUs per sample replicate (mean 119 ± SD 73) and 0–4 OTUs per control replicate (mean 0.3 ± SD 0.6). The total number of *de novo* OTU clusters in the metabarcoding data was 3046, and no attempt was made to link these to formally described species or to BINs.

A total of 36,402 individual arthropods were sorted from the samples (per‐sample mean 809 ± SD 750, range 36–3228). Of these, 32,598 (89.6%) successfully yielded an individual barcode sequence which passed standard quality controls (per‐sample mean 724 ± SD 651, range 32–2538), including an order‐level match to the morphological taxonomic designation provided during sorting. A total of 3636 BINs were recovered from the individual barcoding data, of which 1380 (38%) were annotated at the species level and 2256 (62%) were not. When the same samples were clustered into *de novo* OTUs, the total number of OTUs was 3347. When resolved by individual sequences, samples contained between 10 and 749 BINs (mean 174 ± SD 172) or between 11 and 705 *de novo* OTUs (mean 165 ± SD 161).

**FIGURE 2:**
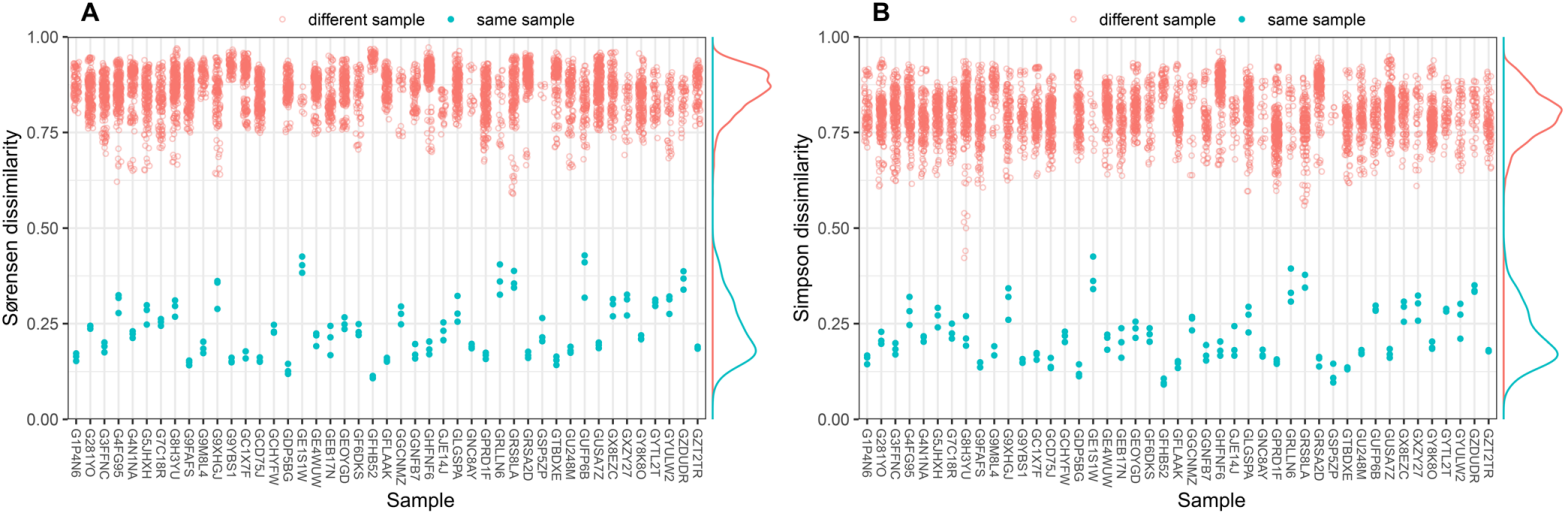
Replicability of metabarcoding samples. Replicability is based on samples processed in three replicates and measured by Sørensen (A) and Simpson (B) dissimilarities. Blue points refer to comparisons between replicates of the same sample, red points to comparisons to other samples.

Metabarcoding turned out to be highly replicable, as species richness was highly consistent among replicates of the same sample whether measured by BINs (*R* ^2^ = 0.997) or by *de novo* OTUs (*R* ^2^ = 0.993). Also, species composition was replicable in the sense that it varied much more between samples than between replicates of the same sample. On average, Sørensen's index of dissimilarity between replicates from the same sample was 0.23 ± 0.08 for mBRAVE and 0.12 ± 0.04 for OptimOTU (see marginal distributions in Figure 2A,B and Figure S1A,B), and Simpson's distances were similar, mean 0.21 ± 0.07 for mBRAVE and 0.15 ± 0.06 for OptimOTU (marginal distributions in Figure 2C,D, Figure S1C,D). In contrast, Sørensen and Simpson dissimilarities between replicates from different samples were substantially higher; 0.86 ± 0.06 and 0.80 ± 0.06 for mBRAVE and 0.91 ± 0.05 and 0.82 ± 0.10 for OptimOTU, respectively (Figure 2, Figure S1).

**FIGURE 3:**
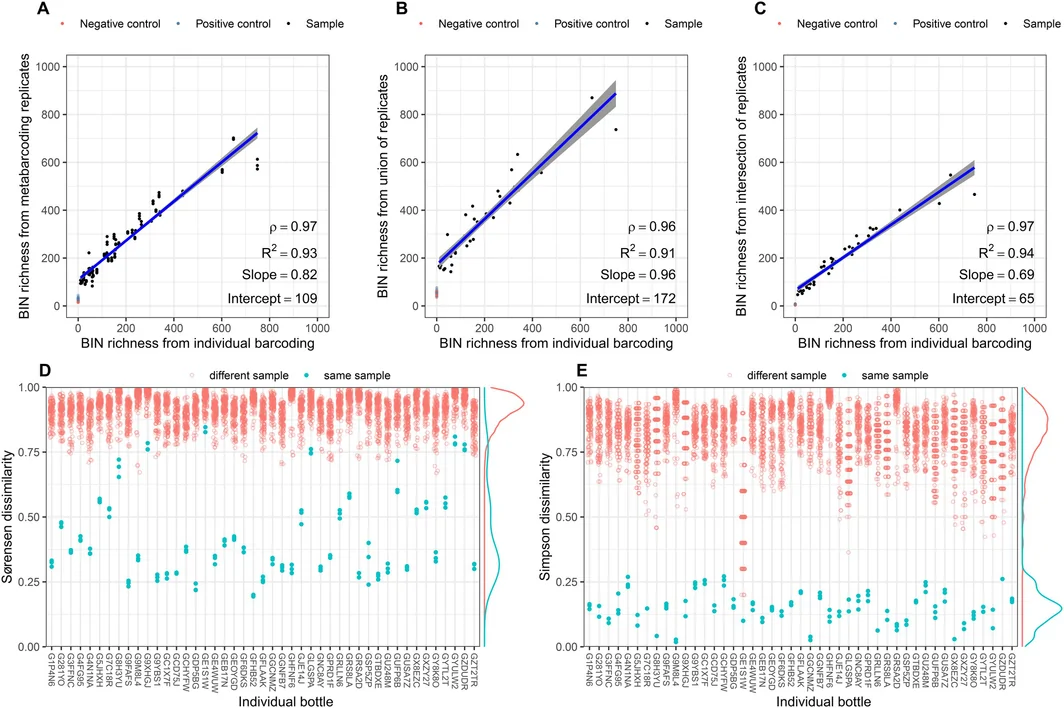
Comparison between individual barcoding vs. metabarcoding. The upper panels compare BIN richness revealed by individual barcoding (x‐axis) to BIN richness revealed by metabarcoding (y‐axis), either counting each replicate separately (A), pooling replicates within each sample (B), or including only BINs present in all three metabarcoding replicates of each sample (C). The blue line shows a linear model fitted to the data, of which key statistics are inserted in the panels. The lower panels show Sørensen (D) and Simpson (E) dissimilarities between individual barcoding vs. metabarcoding for rows of samples. For the individually sequenced data from each sample, we show dissimilarity with all metabarcoding replicates. Blue points refer to comparisons between observations of the same sample, red points to comparisons to other samples.

The OTU richness recovered was also highly consistent among methods, in the sense that a sample perceived to be rich in OTUs by one method was also rich in OTUs by the other (Figure 3, Figure S2). Reference‐based metabarcoding resolved many taxa also from field samples for which we detected a low species richness by individual barcoding (intercept = 172 ± SE 10.9), but for each new BIN resolved by the sequencing of individual insects we detected less than one new BIN by metabarcoding (slope = 0.94 ± SE 0.04). Although the exact intercept and slope values varied, these patterns remained constant when considering each metabarcoding replicate separately (Figure 3A) as when pooling replicates within samples (Figure 3B), or when considering only BINs present in all three replicates of each sample (Figure 3C). Control samples contained fewer BINs than the estimated intercept in all cases, but especially when considering only BINs present in all three control replicates. As the *de novo* pipeline recovered fewer OTUs overall than did the mBRAVE pipeline, estimates of both intercept and slope were correspondingly lower (Figure S2A–C). The curves of OTU richness in metabarcoding vs. individual barcoding (Figure S2) also show a nonlinear trend, suggesting that the ‘true’ intercept may be close to 0, but that OTU detection rates saturate for samples with high OTU richness of individuals.

**FIGURE 4:**
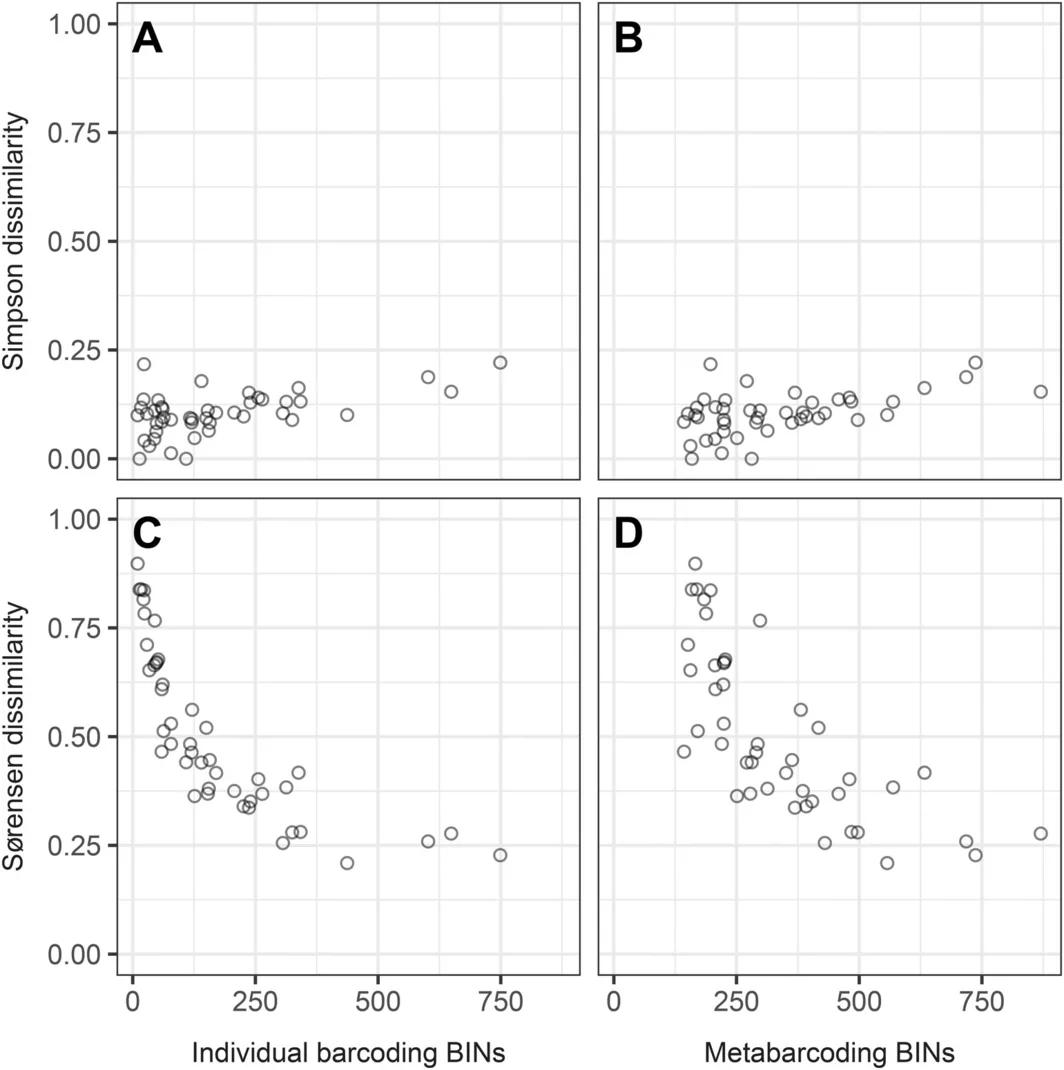
Dependency of individual barcoding vs. metabarcoding dissimilarity on species richness. The panels show Sørensen dissimilarity (upper panels) and Simpson's dissimilarity (lower panels) between community composition detected in individually sequenced and metabarcoded samples, plotted as a function of BIN richness in the sample. Since the two methods detected slightly different numbers of BINs (Figure 3), we plot the same dissimilarity values against BIN richness resolved by either individual barcoding (left‐hand panel) or metabarcoding (right‐hand panel). Each data point is an individual sample, with the dissimilarity between the community detected by the two data types shown by the y‐axis.

Not only richness, but also the community composition recovered was highly consistent among individual barcoding and metabarcoding results for the same sample. This was especially the case with Simpson's dissimilarity, for which dissimilarity between the two methods was on average 0.15 ± 0.06 for mBRAVE and 0.21 ± 0.07 for OptimOTU within samples and 0.82 ± 0.10 for mBRAVE and 0.83 ± 0.09 for OptimOTU between samples. Sørensen's dissimilarity between the methods was on average 0.43 ± 0.17 for mBRAVE and 0.34 ± 0.11 for OptimOTU within samples and 0.91 ± 0.05 for mBRAVE and 0.9 ± 0.06 for OptimOTUbetween samples. The higher values of within‐sample Sørensen dissimilarity (and hence, patterns of nestedness) were especially seen in samples with low OTU richness (especially as measured by individual sequencing, Figure 4 and Figure S3), whereas Simpson's dissimilarity was independent of OTU richness. The consistency between the methods in their estimated community composition was also reflected in the observed correlation between dissimilarity among sample pairs: this was much higher both for Sørensen (mBRAVE *r*: 0.69, OptimOTU *r*: 0.90; both *p* = 0.0001) and Simpson's dissimilarity (mBRAVE *r*: 0.55, OptimOTU *r*: 0.767; both *p* = 0.0001) than expected under random permutation.

**FIGURE 5:**
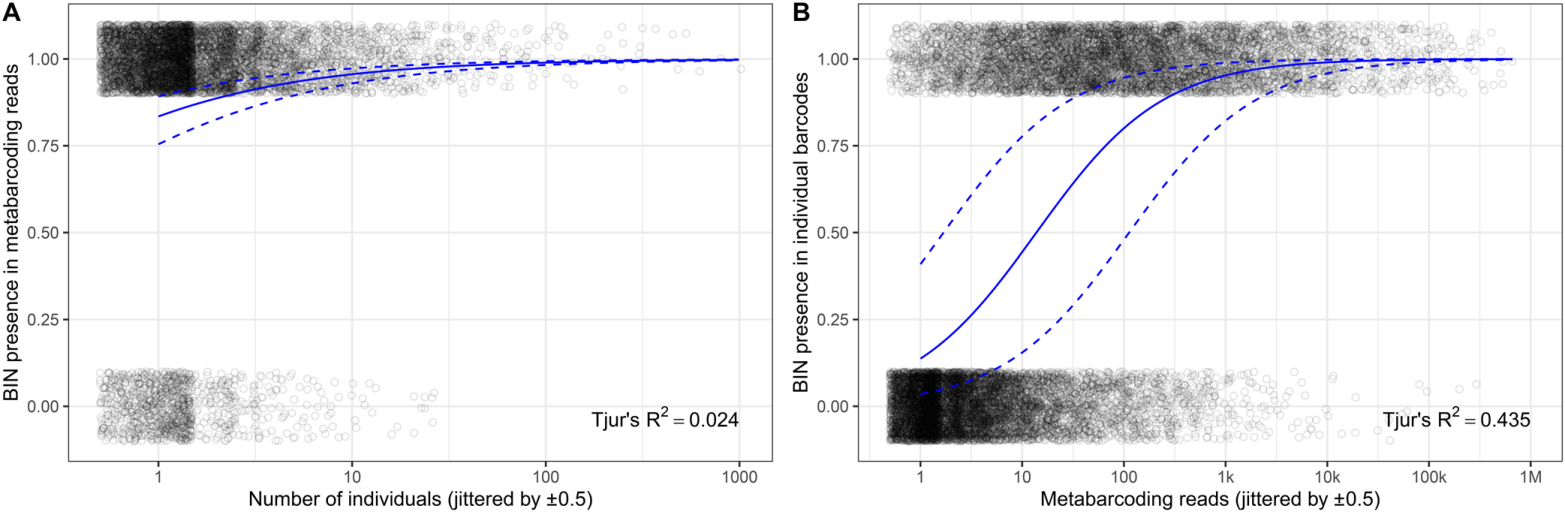
The probability of detecting a BIN present in one type of data in the other type of data. Panel (A) shows metabarcode presence as a function of individual abundance, and panel (B) individual presence as a function of the read abundance of this BIN in the metabarcoding data. The lines show a logistic regression model of Y regressed on X. The logistic regression model also includes log(metabarcoding sequencing depth) as a predictor, and the three lines show the prediction for sequencing depth of 500,000 (continuous line), 250,000 (lower dashed line in A, upper dashed line in B), and 1,000,000 (upper dashed line in A, lower dashed line in B). To resolve individual data points, presence/absence is plotted with jitter around 0 and 1.

The probability of detecting an OTU by metabarcoding was high (83% for mBRAVE, 68% for OptimOTU, both adjusted to sequencing depth of 5 × 10^5^) even for BINs found as a single individual during individual barcoding (Figure 5, Figure S4). From this value, it further increased both with the abundance of the OTU in the individual barcoding data and with increased metabarcoding sequencing depth (Figure 5A, Figure S4A), although these effects had limited explanatory power (Tjur's *R* ^2^ = 0.024 for mBRAVE, 0.083 for OptimOTU). Conversely, an OTU present as a single metabarcoding read was unlikely (10% probability for both pipelines) to be present as an individual (Figure 5B). The probability of presence as an individual increased with the number of metabarcoding reads, reaching an estimated 50% when the number of metabarcoding reads equalled 15 for mBRAVE and 50 for OptimOTU, and reaching 95% when the number of metabarcoding reads equalled 1000 for mBRAVE and 22,000 for OptimOTU. Sequencing depth and OTU read abundance together had some explanatory power for OptimOTU (Tjur's *R* ^2^ = 0.183), but substantially more for mBRAVE (Tjur's *R* ^2^ = 0.435). As expected, the effect of sequencing depth was negative for predicting the presence of an OTU as an individual in the sample.

**FIGURE 6:**
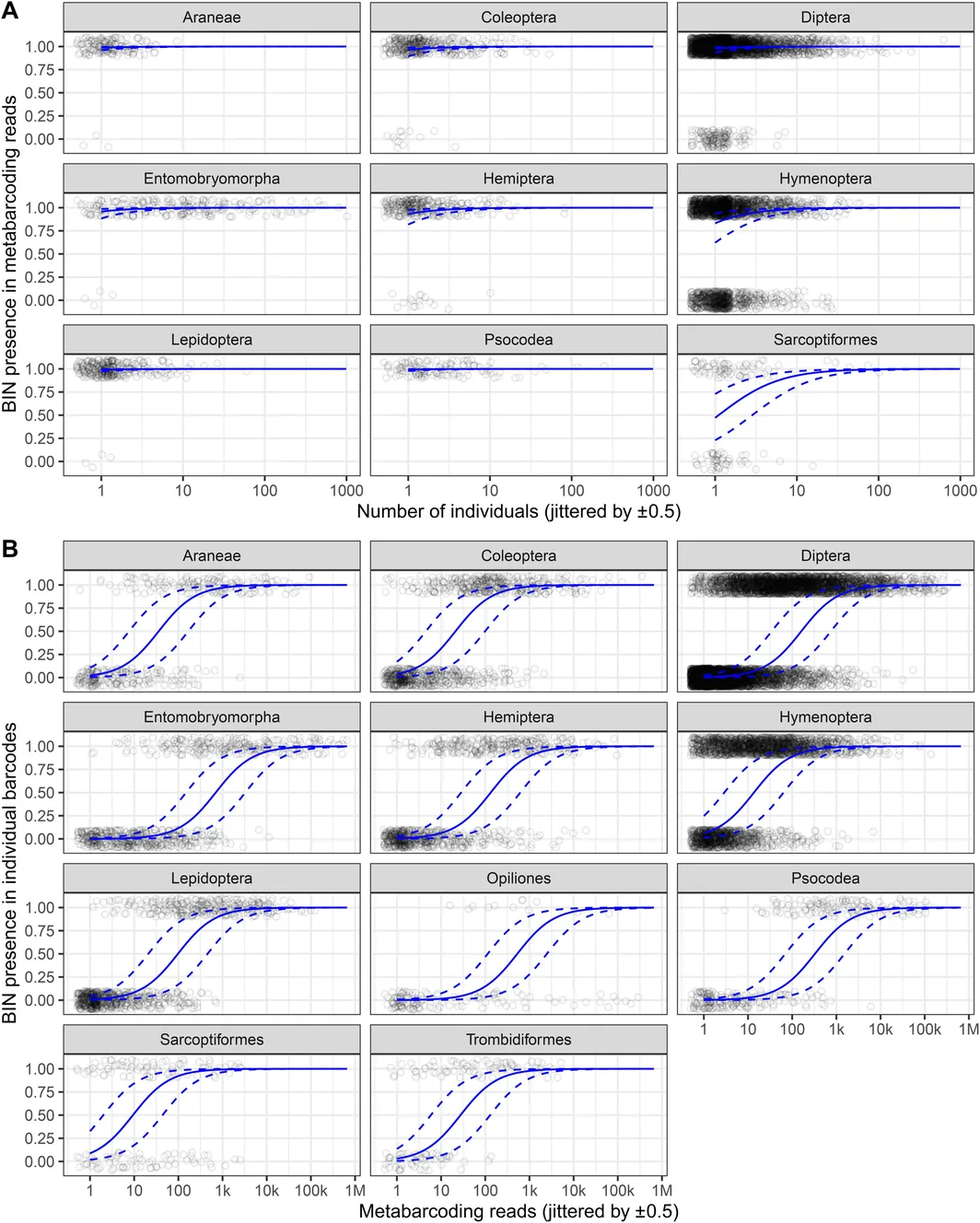
The probability of detecting a BIN present in one type of data in the other type of data. (A) Metabarcode presence as a function of individual abundance, (B) individual presence as a function of the read abundance of this BIN in the metabarcoded data. Here, we break down the general pattern shown in Figure 5 by arthropod orders. The lines show logistic regression models of Y regressed on X, including the order‐level taxonomic random effect, for sequencing depths 250,000 reads (lower dashed line in A, upper dashed line in B), 500,000 reads (solid line in A and B), and 1,000,000 reads (upper dashed line in A, lower dashed line in B). To resolve individual data points, presence/absence is plotted with jitter around 0 and 1.

Adding a taxonomic random effect substantially increased the explanatory power both for metabarcoding presence based on individual abundance (Tjur's *R* ^2^ = 0.46 for mBRAVE, 0.50 for OptimOTU) and for individual presence based on metabarcoding read abundance (Tjur's *R* ^2^ = 0.68 for mBRAVE, 0.55 for OptimOTU). Notably, despite the incompleteness both of the taxonomic annotations and of the taxonomic overlap between samples, the taxonomically informed models performed nearly as well at predicting occurrences in new samples (Tjur's *R* ^2^ = 0.41 and 0.64 for mBRAVE, 0.41 and 0.44 for OptimOTU). At the level of individual arthropod orders, patterns were qualitatively consistent with Figure 5 but showed some idiosyncrasies between given taxa (Figure 6, Figure S5). As examples, almost all Lepidoptera present in the sample were always likely to be detected in the metabarcoding data, whereas small mites were hard to detect (Figure 6A). On the other hand, Opiliones (‘daddy longlegs’) present in the metabarcoded data were rarely found as recognizable individuals (Figure 6B). Addition of a sample‐wise random effect, although it cannot be used to improve prediction for unseen samples, improved explanatory power (Tjur's *R* ^2^ = 0.47 for mBRAVE metabarcoding presence and 0.73 for individual presence; OptimOTU 0.55 and 0.61, respectively), pointing to additional sample‐level characteristics which may influence the detection process.

**FIGURE 7:**
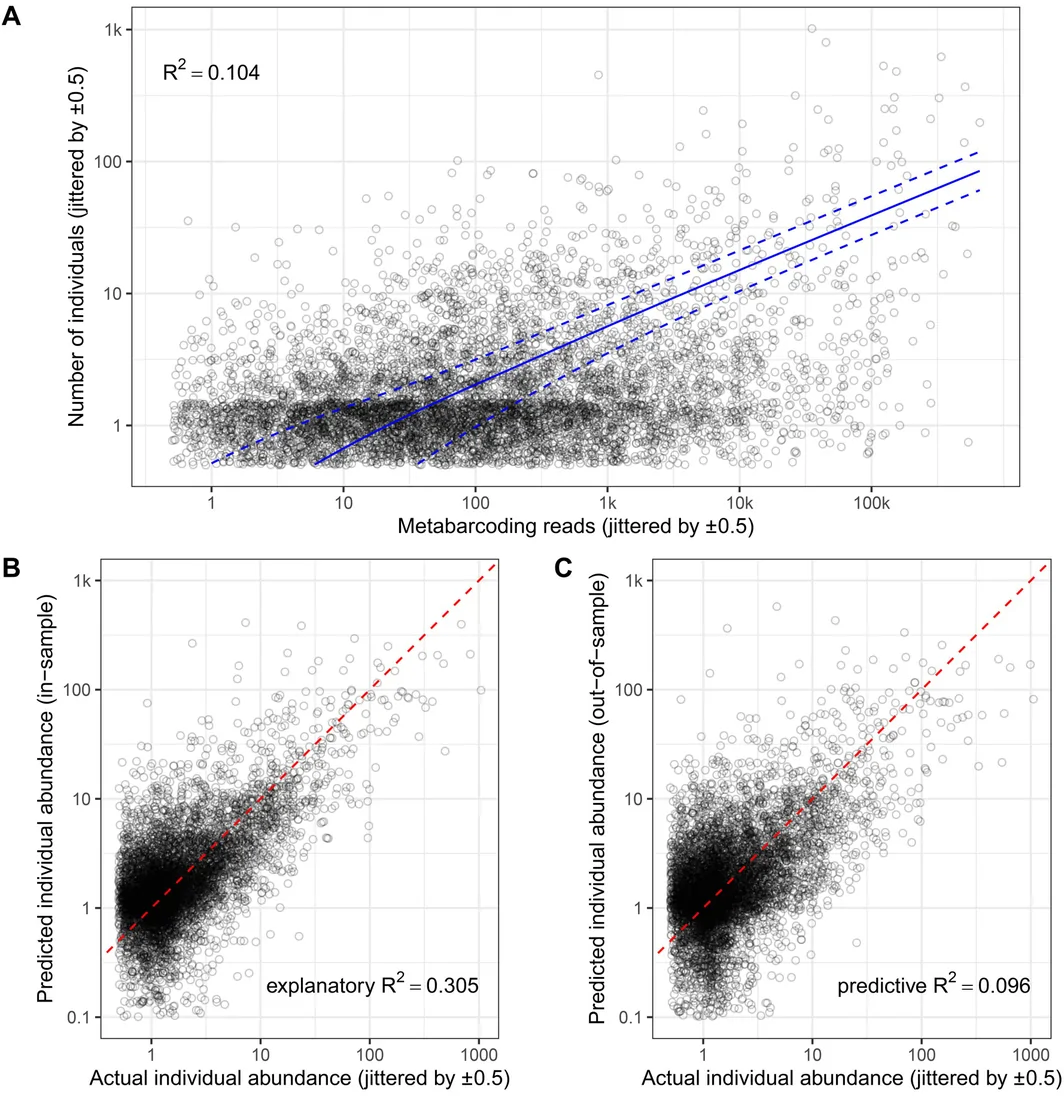
Abundance of individuals as predicted from metabarcoding reads. (A) Observed data along with predictions from a hurdle model fitted to log(number of reads) and log(sequencing depth). Predictions are for sequencing depth of 250,000 reads (upper dashed blue line), 500,000 reads (solid blue line) and 1,000,000 reads (lower dashed blue line). (B) In‐sample predicted vs. actual abundances for a model also including a nested random effect for taxonomic groups at the order, family, and genus levels. The dashed red line is the theoretical 1:1 line for perfect predictions. (C) Out‐of‐sample predicted vs. actual abundances for the same model.

The number of individuals in the sample was predictable by the number of metabarcoding reads, although the pattern was associated with substantial noise (Figure 7, Figure S6). A hurdle model for individual abundance built on top of the model for presence as a function of metabarcoding read number and sequencing depth achieved an *R* ^2^ = 0.10 for mBRAVE (Figure 7A) and 0.11 for OptimOTU (Figure S6A), whereas adding a taxonomic random effect resulted in a major improvement in explanatory power, reaching an overall *R* ^2^ of 0.30 for mBRAVE (Figure 7B) and 0.41 for OptimOTU (Figure S6B). Predictive *R* ^2^ based on sample‐wise leave‐one‐out cross‐validation was 0.10 for mBRAVE (Figure 7C) and 0.06 for OptimOTU (Figure S6C). As for the presence‐only models, further addition of a sample‐wise random effect increased the explanatory power (*R* ^2^ = 0.69 for mBRAVE, 0.75 for OptimOTU), indicating that additional sample‐specific characteristics are important in determining the relationship between metabarcoding and individual abundances.

## 4 Discussion

While metabarcoding is becoming a method of choice for the characterization of arthropod communities, its reliability remains a topic of controversy. In this paper, we have argued that neither sequencing of individuals nor metabarcoding will offer an objective ground truth for the DNA contents of an arthropod sample. To offer a nuanced approach, we asked a series of questions regarding the consistency of metabarcoding replicates, and the consistency of community structure gleaned from individual barcoding and metabarcoding data. We find that metabarcoding in itself is replicable, as different replicates recover similar species richness and composition from the same sample. When compared to individual barcoding, metabarcoding provides a similar impression of relative differences in community structure, as estimates of species richness are highly correlated among methods, and as relational dissimilarity between samples prevails among data types. Importantly, the differences in species composition perceived between sample types do depend on BIN richness in the sample, but this relationship seems due to nestedness rather than turnover. Our most important findings relate to the predictability of BIN occurrence in one sample type by the other: here, any BIN recovered from individually sequenced arthropods was detected with high probability in the metabarcoding data, and any BIN found in high read abundances in the metabarcoding data was likely to be found as an individual. Compared to this high predictability of presence/absence, predictions regarding abundances proved less precise, but accounting for differences among taxa improved predictions substantially. Below, we will discuss each finding in turn.

### 4.1 Metabarcoding Is Qualitatively Replicable

Different replicates of the same samples recovered much of the same species composition. An average of 77% of the BINs detected in any one replicate was shared with any other replicate from the same sample. When combined with the high probability (80%) that any BIN present as an individual in the sample was also detected in the metabarcoding sequence yield, these results attest to the replicability of metabarcoding assays and the robustness of the descriptors attained. Our current results are thus consistent with those of Van Den Bulcke et al. (2023), who showed high consistency in species numbers, Shannon indices and Inverse Simpson indices recovered for samples of marine benthos—even when the samples were analysed by different laboratories in a ring test.

Having said that, we fully subscribe to the general recommendation that metabarcoding should be run as replicates (Zinger et al. 2019), providing not only more data but also enabling quality control. Since chance variation will generate differences between replicates, more is probably better (Leray and Knowlton 2017; Shelton et al. 2023). However, a counterpoint is offered by Smith and Peay (2014), who suggest that many studies which recommend PCR replicates are confounding replication with total sequencing depth. This confounding occurs as recommendations of replication are based on simply finding more of the low‐read‐abundance species from a mock community by sequencing 10 replicates, whereas no test is offered for whether the same study would have produced the same result by sequencing a single replicate 10 times deeper. The latter approach is arguably cheaper and faster, but allows fewer opportunities for quality control. It is also worth noting that the sequencing depth of individual replicates in this study (mean 214,000 reads) is substantially larger than used in many earlier studies, including Smith and Peay (pools of 1–16 replicates rarefied to 500 reads for 454, 38,000 reads for Illumina) or Leray and Knowlton (mean 65,000 reads per replicate), and this may be an important factor for replicability.

When samples are sequenced in replicates, the question emerges whether the data from individual replicates should be retained as nested data points of the same sample or pooled for analysis (as we have done in our subsequent analyses). This will depend on the modelling approach chosen, whereas for the current argument, the key finding is that individual replicates will be largely consistent, and the core species pool will be shared between individual replicates. That such consistency is the rule is also shown by another consideration: as a part of quality control, CBG tends to omit taxa found in less than two out of three replicates, but this will typically result in the removal of < 15% of BINs (Evgeny V. Zakharov, unpublished). This is supported by our results, which found that, due to the high sensitivity of the reference‐based approach used by the mBRAVE pipeline, some potential for (cross‐)contamination remains. Such contamination was evidenced by the detection of BINs in control samples, but this effect was almost eliminated by restricting to BINs detected in all three replicates. The OptimOTU pipeline includes more filtering stages, which reduces sensitivity to both contaminants and target OTUs, so it may be more appropriate to pool the replicates without additional filtering. However, the exact balance between sensitivity to true sequences and precision to avoid artefactual and contaminant sequences is expected to depend not only on the choice of overall bioinformatics workflow but also on specific settings (Eisele et al. 2026).

### 4.2 Individual Barcoding and Metabarcoding Provide the Same Impression of Relative Differences in Community Structure

Our comparison of community metrics between metabarcoded and individually barcoded samples revealed a general agreement in terms of relative differences between the samples: a sample comparatively rich in OTUs scored by one method was also rich in OTUs scored by the other, and a sample dissimilar from other samples within one data type was also dissimilar within the other data type. This attests to the reliability of both methods, and to their partial interchangeability with each other. What is more, the finding that differences in species composition reflect nestedness rather than turnover provides directional evidence that potential biases are not causing metabarcoding to lose large numbers of taxa detectable by individual barcoding.

In showing that metabarcoding can reliably recapture the contents of insect individuals visible in an arthropod sample, our findings support those of Remmel et al. (2024). These authors compared the arthropod contents of 12 Malaise samples as recovered by metabarcoding to the contents scored by morphological identification by taxonomic experts. Of 252 species identified in total, 54.8% were identified by both methods, whereas only 21.4% and 19.8% were detected exclusively by metabarcoding or morphology, respectively. Matching our current results, these authors also found good correspondence between methods in terms of total taxonomic richness per sample. In terms of community composition, our results echo the original findings of Yu et al. (2012). Using early 454 pyrosequencing techniques, these authors demonstrated that metabarcoding allows for precise estimation of both alpha diversity and pairwise community dissimilarity (beta diversity). Together, our studies thus show that metabarcoding will be informative regarding the composition of local insect communities and differences between them, and that the patterns resolved are comparable to those determined through traditional, morphological identification or through individual barcoding.

In evaluating the consistency of metabarcoding data for purposes of biodiversity discovery, community comparisons, ecological monitoring and testing of topical theories in community ecology, the question of what is ‘consistent enough’ will clearly emerge. Here, the answer will depend on what the data are used for. Showing that two methods each produce internally consistent measurements does not establish that they are interchangeable or suitable for decision‐making. Whether, for example, a correlation coefficient of *r* = 0.69 of between‐sample Sorensen dissimilarities is satisfactory or not will depend on the question asked. For example, they could be sufficient for community composition differences between disparate sites but insufficient for biomonitoring. Here what matters is whether the magnitude of disagreement between methods is compatible with detecting fine‐scale ecological change over time and space. This is for the user to evaluate.

In evaluating the repeatability of metabarcoding results, it is perhaps sobering to put the results in the context of other sources of variation. The consistency between metabarcoding replicates and data types here observed is very much higher than the consistency in catches between traps placed only a few metres apart. In vivid illustration of this, the addition of even two additional traps within a site can increase the species richness observed by 40% (Matthews and Matthews 1983). In the grasslands of Canada (Steinke et al. 2021), traps placed just 18 m apart differed dramatically in composition, and in tropical forests, trap catches < 400 m apart were almost completely different (Chimeno et al. 2023). Variation between methods is thus no larger than the effects of shifting the trap's position by a meter, or turning it a few degrees (Fraser et al. 2008; Irvine and Woods 2007). In this light, the level of consistency between different methods for resolving sample contents seems high compared to sources of variation affecting whether a species even reaches the sample. What we suggest is that we should next turn our attention from characterizing the sample once in the bottle to the many processes affecting how an insect ends up there. It is the surrounding community that we wish to make inferences about, and accurately modelling the relation between the sample and this reference community will seem like the next key step (Rodriguez et al. 2026).

### 4.3 Metabarcoding Reveals Many More Taxa Than Individual Barcoding

Two differences stand out between the metabarcoding and individual sequencing data here examined: First, the metabarcoded samples processed by the more sensitive reference‐based pipeline showed substantially higher species richness, with an average excess of 172 BINs for any one BIN detected by sequencing of individual insects (Figure 3B). Second, starting from this initial excess (i.e., the intercept+1 in Figure 3B), metabarcoding data accumulated 0.96 new BINs for each new BIN detected in individually sequenced samples (i.e., the slope in Figure 3B).

Importantly, the higher BIN richness observed in metabarcoding data refers to arthropod BINs alone, since all other phyla were pruned from the data (see Methods). Thus, the higher BIN richness observed in metabarcoding data cannot be attributed to the resolution of a wide array of protists, bacterial symbionts, etc. from the arthropods by metabarcoding data (Łukasik and Kolasa 2024). Instead, a metabarcoded sample will reveal the presence of hundreds of *arthropod* species beyond the individuals trapped in the collecting bottle. At the same time, the surplus of species observed in metabarcoding data cannot be entirely attributed to cross‐contamination between samples, since both positive and negative controls show substantially fewer BINs than the intercept. This is most clear when using the more stringent method for combining replicates, where only BINs detected in all three replicates are counted for the sample (Figure 3C), in which case the control samples have almost no BIN detections (median 3, range 0–9), while the field samples still show an intercept of 65 species. However, note that this method entails a trade‐off, since the slope of 0.69 indicates that many species present as individuals are being missed.

All things considered, the added set of species recorded by metabarcoding, especially after removing BINs not found in all replicates, seems attributable to a strong imprint of biotic interactions on the DNA content of an arthropod sample. Beyond the arthropods recovered from individually sequenced samples, each individual seems to carry a wide array of DNA residues from past encounters with other arthropods. We shall return to this issue below, in the context of predicting the probability of species occurrence in one type of data by BIN abundance in the other type of data.

When it comes to the slope of less than one (0.94) new BIN added to metabarcoding data for every new BIN detected by the sequencing of individuals, we note that this estimate is qualitatively consistent with a simple consideration: while arthropod communities are diverse, they are not limitless in their composition. Thus, towards more species‐rich samples, the increase in the Sørensen index decelerates (Figure 4). This may be due to much of the additional diversity (in the form of gut contents and surface residues) already being recorded from smaller samples, and richer samples thus showing higher redundancy with less species‐rich samples (i.e., nestedness)—as there are simply less species left to add.

In terms of what BINs are resolved by the two data types, the difference in patterns between Sørensen and Simpson dissimilarities sheds light on the underlying phenomena (Figure 4): while Sørensen dissimilarity increases among data types with increasing BIN richness in the target sample, no such relation was detected for Simpson dissimilarities (cf. Figure 4A,B vs. C,D). Since Sørensen dissimilarity is sensitive to differences in species richness but Simpson dissimilarities are not, the pattern in the former seems largely attributable to nestedness, i.e., to differences in BIN richness resolved by the two methods. Thus, metabarcoding seems to add further species beyond the core group occurring as intact individuals in the sample, whereas any BIN occurring as an intact individual is detected with a high probability (Figure 5A).

### 4.4 We Can Predict the Probability of Species Occurrence in One Type of Data by Its Abundance in the Other Type of Data

The probability of detecting a BIN present in one type of data in the other type of data, too, varied substantially with its abundance. We note that this effect is completely different from an effect of sequencing depth alone, since sample‐to‐sample variation in read depth was already captured by entering log(sequencing depth) as a covariate. Both before and after adjusting for this obvious effect, BINs more abundant in one type of data were more frequently detected in the other (Figure 5). Nonetheless, the relationship differed substantially between when we used abundance among individuals sequenced to predict presence in metabarcoding data (Figure 5A) and when we used metabarcoding read abundance to predict presence among sequenced individuals (Figure 5B): even BINs present as single individuals in the sample were detected in the metabarcoding data with a probability of 83%, and this probability further approached one for abundant taxa (Figure 5A). In contrast, BINs detected in low metabarcoding read numbers were unlikely to be found as recognizable individuals (*p* ≅ 10%; Figure 5A), whereas this probability quickly rose above 95% for BINs encountered in more than 1000 reads, and approached one for BINs encountered in more than 10,000 reads (Figure 5B).

At the level of individual arthropod orders, these patterns were qualitatively consistent, with some idiosyncrasies between given taxa (Figure 6). That small mites are hard to detect is only to be expected, given that they include tiny amounts of DNA and can simultaneously be enclosed in sclerotized shells (Elo 2019). Opiliones, on the other hand, feature extremely long and slender appendages, which are prone to breaking. Whether the frequent occurrence of daddy longlegs in metabarcoded data (Figure 6B) will reflect that they often climb into the collecting part of the trap, but then escape after losing some appendage, and/or whether Opiliones are frequently consumed by predatory species later recovered in the traps cannot be established here—but both seem likely. General variation in the detectability of different taxa was also reflected by the fact that adding a random effect of taxonomy will improve model fit for both presence/absence and abundances (see below).

Overall, these findings show that any BIN collected as an entire individual in the bottle will be detected in metabarcoding data with a high probability, conditional on adequate sequencing depth. Taxa rare in the sample will naturally be slightly harder to detect, as also found by Remmel et al. (2024). At the same time, our findings again attest to an excessive number of BINs *not* occurring as full individuals in the data, and thus *not* detectable by individual barcoding—unless each individual is deeply sequenced and thereby essentially metabarcoded. The different relation uncovered when we used abundance among individuals sequenced to predict presence in metabarcoding data reveals a strong signature of biotic interactions on metabarcoded samples. As all the additional BINs will be arthropods, but arthropods not detectable as individuals, a major part is likely to emanate from gut contents. Here, both primary predation and even ‘secondary predation’ (where a predator is consumed by another one after consuming a prey species; Tercel et al. 2021) seem likely to contribute. Malaise traps coupled with high throughput sequencing will thus reveal an even wider view of the local fauna than its content of insect individuals. Importantly, a species present in the gut content or on the surface of another, or even as free airborne environmental DNA, will seem no less a member of the local community than a species present as an individual—and our current estimates suggest that the number of such species per physically detectable species ranges in the hundreds. Whether the sequencing of individual insects or metabarcoding will then constitute the preferred method (Chua et al. 2023) will depend on the question asked, but clearly, the two should be seen as mutually complementary rather than conflicting methods.

### 4.5 We Can Partly Predict the Abundance of Individuals From Metabarcoding Read Numbers

The level of predictability was substantially lower for abundance than presence, with *R* ^2^ values varying around 0.1, depending on the model applied. This is only to be expected, given that some of the taxa are very large and some are small. Indeed, Iwaszkiewicz‐Eggebrecht et al. (2026) recently showed that quantification from read numbers can be substantially improved by using biological spike‐ins and more explicitly derived correction factors. In the current context, substantial variation in the relation between individual abundance and metabarcoding read numbers is revealed by substantial model improvement when adding a taxonomically structured random effect—as echoing the findings regarding presence/absence analyses (see above). There is thus no question that quantification of insect abundances from metabarcoding data comes with both major uncertainty and taxon‐specific biases. At the same time, the general relationship here observed *and* the improvements made possible by spike‐ins (here lacking) and more in‐depth statistical modelling (Iwaszkiewicz‐Eggebrecht et al. 2026) offer every cause for optimism in terms of recovering abundance information from metabarcoding data.

## 5 Conclusions

Overall, our results provide a hope‐inspiring view on metabarcoding as a method for generating reliable, verifiable and efficient insights into arthropod communities. The vast majority of taxa present will be detected by metabarcoding, given sufficient sampling depth and adequate lab protocols. In the current case, conditional on the specific methods applied, this probability exceeded 80% even when a single individual was present. As such, our findings suggest that metabarcoding can actually deliver on the grand promise given by Ji et al. (2013) over a decade ago. At the same time, it is clear that metabarcoding involves some level of stochastic variation. Based on our results, the level of this stochastic variation is relatively small, and its effect can be mitigated by replication of the metabarcoding process. In our view, the most important take‐home message from our study is that metabarcoding will resolve community features *beyond* those captured by individual barcoding, and that the two approaches should be seen as complementary rather than alternative to each other.

## Funding

O.O. was funded and the contributions of B.F. supported by the Research Council of Finland (grant no. 336212 and 345110) and the European Union (the HORIZON‐CL6‐2021‐BIODIV‐01 project 101059492). The contributions of T.R. and O.O. were funded by the European Research Council (ERC) under the European Union's Horizon 2020 research and innovation programme (grant agreement No 856506; ERC‐synergy project LIFEPLAN). Barcoding and metabarcoding of the samples were funded by the Swedish Environmental Protection Agency (agreement 225‐20‐002 with T.R.). This work was enabled in part by awards from the New Frontiers in Research Fund (NFRFT‐2020‐00073) and the Canadian Foundation for Innovation (MSI 42450) that sustain core operations at the Centre for Biodiversity Genomics.

## Conflicts of Interest

The authors declare no conflicts of interest.

## Supporting information


**Figure S1:** Replicability of metabarcoding samples, following *de novo* clustering of metabarcoding sequences. Replicability is based on samples processed in three replicates and measured through measured by Sørensen (A) and Simpson (B) dissimilarities. Blue points refer to comparisons between replicates of the same sample, red points to comparisons to other samples. This figure directly replicates Figure 2 of the main text, but differs in the bioinformatic approach adopted.

**Figure S2:** Comparison between individual barcoding vs. metabarcoding, following *de novo* clustering of metabarcoding sequences. The upper panels compare OTU richness revealed by individual barcoding (x‐axis) to BIN richness revealed by metabarcoding (y‐axis), either counting each replicate separately (A), pooling replicates within each sample (B), or including only OTUs present in all three metabarcoding replicates of each sample (C). The line shows a linear model fitted to the data, of which key statistics are inserted in the panels. The lower panels show Sørensen (D) and Simpson (E) dissimilarities between individual barcoding vs. metabarcoding for rows of samples. For the individually sequenced data from each sample, we show dissimilarity with all metabarcoding replicates. Blue points refer to comparisons between observations of the same sample, red points to comparisons to other samples. This figure directly replicates Figure 3 of the main paper, but differs in the bioinformatic approach adopted.

**Figure S3:** Dependency of individual barcoding vs. metabarcoding dissimilarity on species richness, following *de novo* clustering of metabarcoding sequences. The panels show Sørensen dissimilarity (upper panels) and Simpson's dissimilarity (lower panels) between community composition detected in individually sequenced and metabarcoded samples, plotted as a function of OTU richness in the sample. Since the two methods detected slightly different numbers of OTUs (Figure S1), we plot the same dissimilarity values against OTU richness resolved by either individual barcoding (left‐hand panel) or metabarcoding (right‐hand panel). Each data point is an individual sample, with the dissimilarity between the community detected by the two data types shown by the y‐axis. This figure directly replicates Figure 4 of the main paper, but differs in the bioinformatic approach adopted.

**Figure S4:** The probability of detecting an OTU present in one type of data in the other type of data, following *de novo* clustering of metabarcoding sequences. Panel (A) shows metabarcode presence as a function of individual abundance, and panel (B) individual presence as a function of the read abundance of this OTU in the metabarcoding data. The lines show a logistic regression model of Y regressed on X. The logistic regression model also includes log(metabarcoding sequencing depth) as a predictor, and the three lines show the prediction for sequencing depth of 500,000 (continuous line), 250,000 (lower dashed line in A, upper dashed line in B), and 1,000,000 (upper dashed line in A, lower dashed line in B). To resolve individual data points, presence/absence is plotted with jitter around 0 and 1. This figure directly replicates Figure 5 of the main paper, but differs in the bioinformatic approach adopted.

**Figure S5:** The probability of detecting an OTU present in one type of data in the other type of data, following *de novo* clustering of metabarcoding sequences. (A) Metabarcode presence as a function of individual abundance, (B) individual presence as a function of the read abundance of this OTU in the metabarcoded data. Here, we break down the general pattern shown in Figure S4 by arthropod orders. The lines show logistic regression models of Y regressed on X, including the order‐level term of a taxonomic random effect, for sequencing depths 250,000 reads (lower dashed line in A, upper dashed line in B), 500,000 reads (solid line in A and B), and 1,000,000 reads (upper dashed line in A, lower dashed line in B). To resolve individual data points, presence/absence is plotted with jitter around 0 and 1. This figure directly replicates Figure 6 of the main paper, but differs in the bioinformatic approach adopted.

**Figure S6:** Abundance of individuals as predicted from metabarcoding reads, following *de novo* clustering of metabarcoding reads. (A) Observed data along with predictions from a hurdle model fitted to log(number of reads) and log(sequencing depth). Predictions are for sequencing depth of 250,000 reads (upper dashed blue line), 500,000 reads (solid blue line) and 1,000,000 reads (lower dashed blue line). (B) In‐sample predicted vs. actual abundances for a model also including a nested random effect for taxonomic groups at the order, family, and genus levels. The dashed red line is the theoretical 1:1 line for perfect predictions. (C) Out‐of‐sample predicted vs. actual abundances for the same model. This figure directly replicates Figure 7 of the main paper, but differs in the bioinformatic approach adopted.

**Table S1:** Summary statistics on the overall data, following de novo clustering of metabarcoding sequences. Shown are the numbers of samples of different type and number of reads passing each step in the bioinformatic pipeline. This Table directly replicates Table 1 of the main paper, but differs in the bioinformatic approach adopted. Thus, the set of steps is also different.

## Data Availability

The primary data used in this study were published as a data release paper by Orsholm et al. (2026). Processed data including community tables, dissimilarity matrices, and fitted models, as well as the R code to generate them and a copy of the ENA accession numbers for raw reads used in this study will be made available upon publication at Zenodo at https://doi.org/10.5281/zenodo.20602082.
