# Supplementary material for: Is There a Fly in My Soup? To What Extent Do Metabarcoding and Individual Barcoding Tell the Same Story?
Source: Mol Ecol Resour. 2026 Sep 11;26(7):e70195. doi: 10.1111/1755-0998.70195 (PMC13564098; doi:10.1111/1755-0998.70195)
Supplement: Supplementary file 2 [file MEN-26-e70195-s002.pdf]

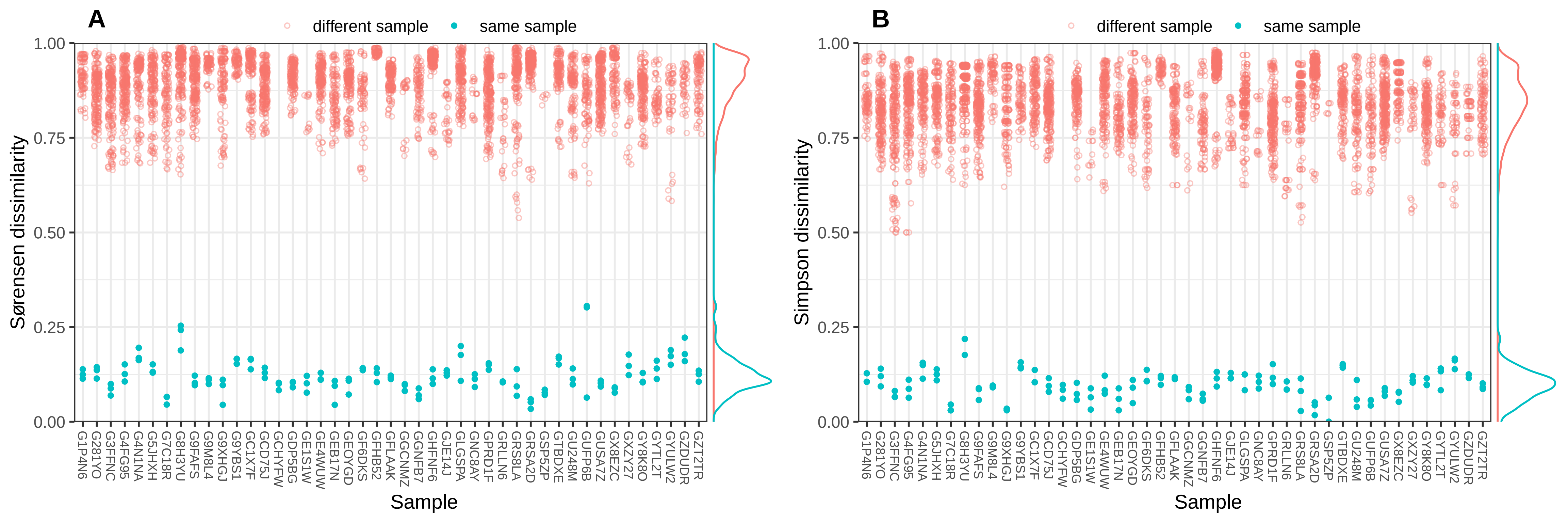
A
different sample
same sample
Sørensen dissimilarity
1.00
0.75
0.50
0.25
0.00
G1P4N6
G281YO
G3FFNC
G4FG95
G4N1NA
G5JHXH
G7C18R
G8H3YU
G9FAFS
G9M8L4
G9XHGJ
G9YBS1
GC1X7F
GCD75J
GCHYFW
GDP5BG
GE1S1W
GE4WUW
GEB17N
GEOYGD
GF6DKS
GFHB52
GFLAAK
GGCNMZ
GGNFB7
GHFNF6
GJE14J
GLGSPA
GNC8AY
GPRD1F
GRLLN6
GRS8LA
GRSA2D
GSP5ZP
GTBDXE
GU248M
GUFP6B
GUSA7Z
GX8EZC
GXZY27
GY8K8O
GYTL2T
GYULW2
GZDUDR
GZT2TR
Sample
B
different sample
same sample
Simpson dissimilarity
1.00
0.75
0.50
0.25
0.00
G1P4N6
G281YO
G3FFNC
G4FG95
G4N1NA
G5JHXH
G7C18R
G8H3YU
G9FAFS
G9M8L4
G9XHGJ
G9YBS1
GC1X7F
GCD75J
GCHYFW
GDP5BG
GE1S1W
GE4WUW
GEB17N
GEOYGD
GF6DKS
GFHB52
GFLAAK
GGCNMZ
GGNFB7
GHFNF6
GJE14J
GLGSPA
GNC8AY
GPRD1F
GRLLN6
GRS8LA
GRSA2D
GSP5ZP
GTBDXE
GU248M
GUFP6B
GUSA7Z
GX8EZC
GXZY27
GY8K8O
GYTL2T
GYULW2
GZDUDR
GZT2TR
Sample
