# Supplementary material for: Is There a Fly in My Soup? To What Extent Do Metabarcoding and Individual Barcoding Tell the Same Story?
Source: Mol Ecol Resour. 2026 Sep 11;26(7):e70195. doi: 10.1111/1755-0998.70195 (PMC13564098; doi:10.1111/1755-0998.70195)
Supplement: Supplementary file 3 [file MEN-26-e70195-s003.pdf]

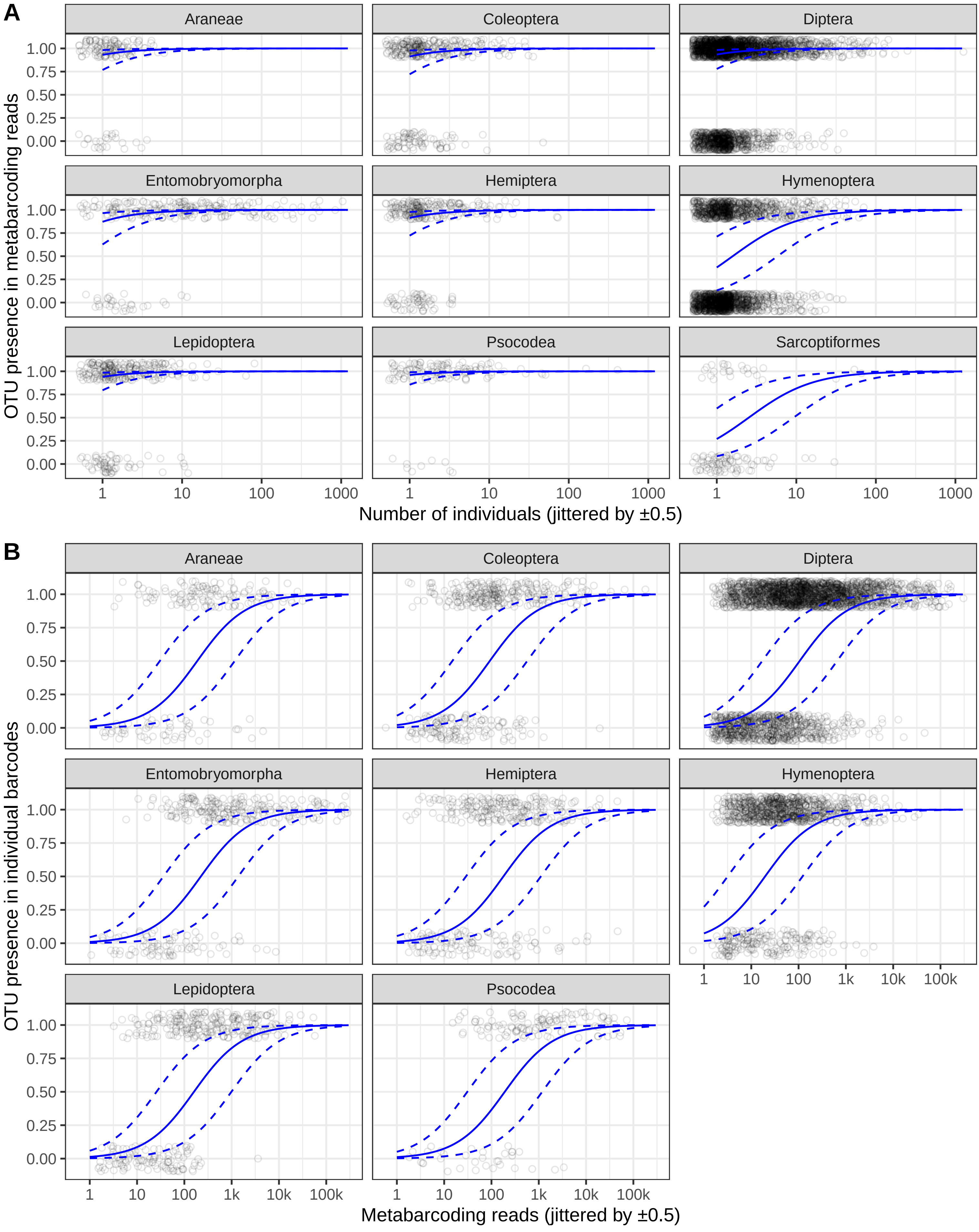
A
Araneae
Coleoptera
Diptera
Entomobryomorpha
Hemiptera
Hymenoptera
Lepidoptera
Psocodea
Sarcoptiformes
OTU presence in metabarcoding reads
1.00
0.75
0.50
0.25
0.00
1
10
100
1000
Number of individuals (jittered by ±0.5)
B
Araneae
Coleoptera
Diptera
Entomobryomorpha
Hemiptera
Hymenoptera
Lepidoptera
Psocodea
OTU presence in individual barcodes
1
10
100
1k
10k
100k
Metabarcoding reads (jittered by ±0.5)
