# Supplementary material for: Is There a Fly in My Soup? To What Extent Do Metabarcoding and Individual Barcoding Tell the Same Story?
Source: Mol Ecol Resour. 2026 Sep 11;26(7):e70195. doi: 10.1111/1755-0998.70195 (PMC13564098; doi:10.1111/1755-0998.70195)
Supplement: Supplementary file 4 [file MEN-26-e70195-s004.pdf]

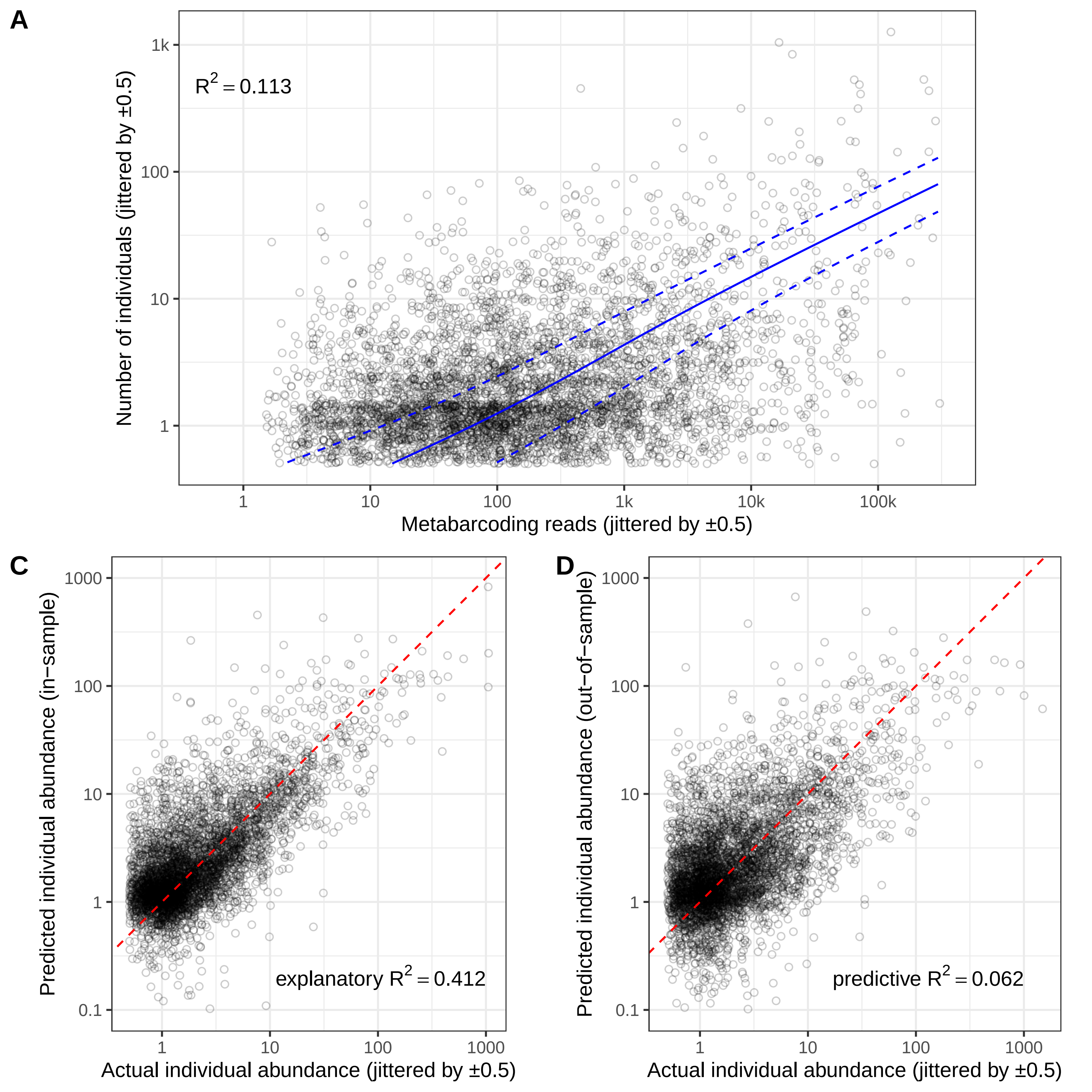
A
$R^2 = 0.113$
Number of individuals (jittered by ±0.5)
Metabarcoding reads (jittered by ±0.5)
1
10
100
1k
10k
100k
C
Predicted individual abundance (in−sample)
explanatory $R^2 = 0.412$
Actual individual abundance (jittered by ±0.5)
0.1
1
10
100
1000
D
Predicted individual abundance (out−of−sample)
predictive $R^2 = 0.062$
Actual individual abundance (jittered by ±0.5)
