# Supplementary material for: Is There a Fly in My Soup? To What Extent Do Metabarcoding and Individual Barcoding Tell the Same Story?
Source: Mol Ecol Resour. 2026 Sep 11;26(7):e70195. doi: 10.1111/1755-0998.70195 (PMC13564098; doi:10.1111/1755-0998.70195)
Supplement: Supplementary file 6 [file MEN-26-e70195-s007.pdf]

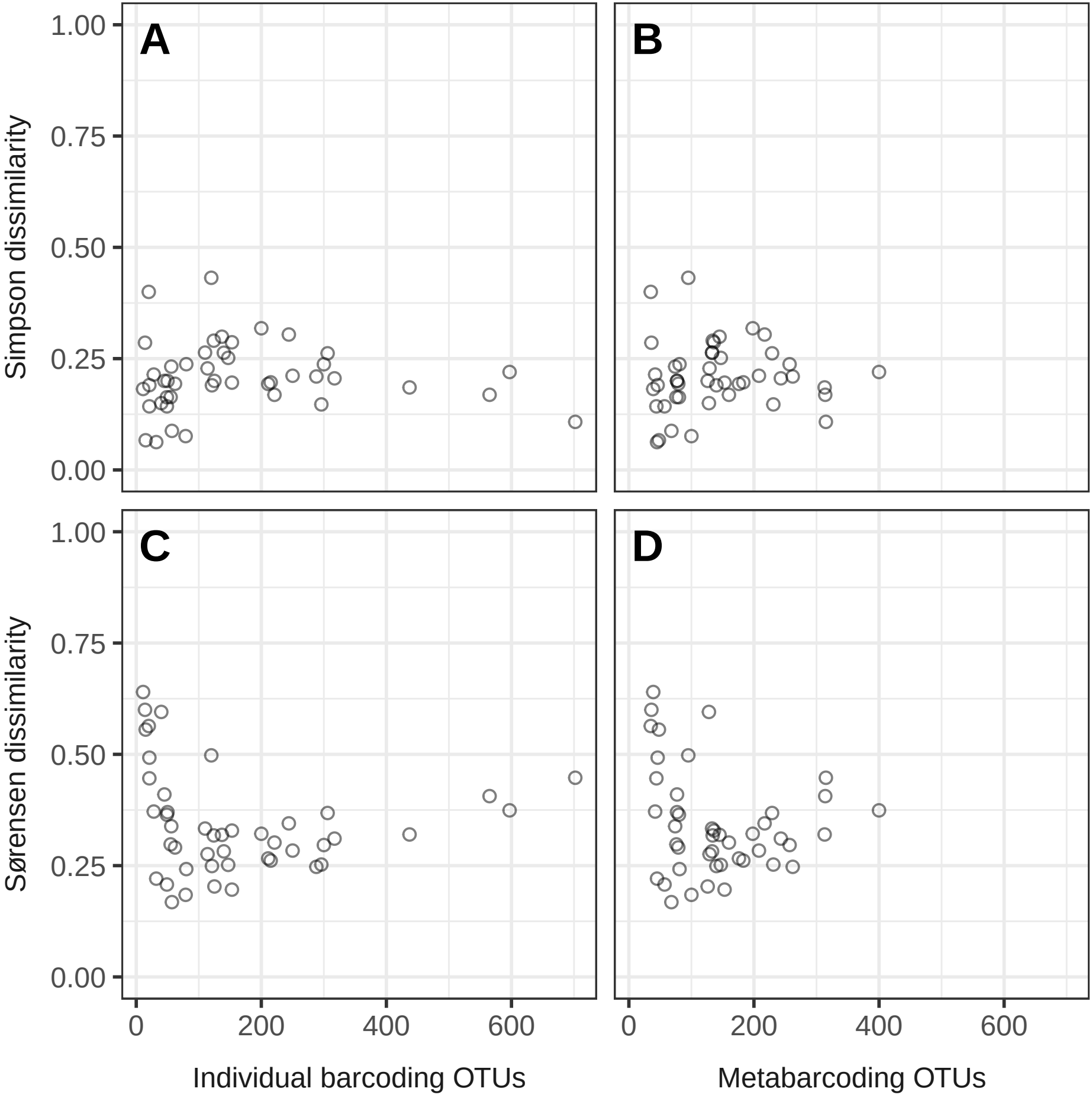

A
B
C
D
Simpson dissimilarity
Sørensen dissimilarity
1.00
0.75
0.50
0.25
0.00
0
200
400
600
Individual barcoding OTUs
Metabarcoding OTUs
