# Supplementary material for: Is There a Fly in My Soup? To What Extent Do Metabarcoding and Individual Barcoding Tell the Same Story?
Source: Mol Ecol Resour. 2026 Sep 11;26(7):e70195. doi: 10.1111/1755-0998.70195 (PMC13564098; doi:10.1111/1755-0998.70195)
Supplement: Supplementary file 7 [file MEN-26-e70195-s006.docx]

# Supplement S1. An alternative workflow based on *de novo* clustering of sequences

Incomplete databases are some of the major limitations of metabarcoding. As a key challenge to real-life metabarcoding exercises, any researcher will thus be faced with the choice of whether to trust novel sequences not observed in the reference library as traces of true taxa or as spurious sequence variants generated by the methods employed. From this perspective, our current experimental setting provides a best-case scenario: we can explicitly use a reference-based approach, since the BINs represented by real individuals can be added to the reference set to which metabarcoding reads are compared (see Methods in main text). Nonetheless, the current scenario will depart from any analysis based on metabarcoding data alone, since here there is no separate “cheat sheet” regarding what taxa to expect. Thus, the researcher will have to adopt a series of steps for inferring what sequence variants are real and which are not. Here, rare sequence variants offer a particular challenge, since occasional sequencing errors are more likely to generate rare sequence variants rather than dominant ones. As a result, most bioinformatic pipelines will treat singletons and low-abundance variants with caution and often exclude them per default.

This is where the strength of our reference-based approach comes into play. As we know the real BINs included in the sample (as individuals), we do not need to filter them out. This avoids the case where biologically real BINs are treated as sequencing errors. If they were, we would not know that they were there in low abundance counts, since we would intentionally have deleted them. As a result, we would remove the far-left end of any axis showing metabarcoding reads.

While the above arguments will illustrate the strengths of our current approach, they also raise a concern: would the current conclusions also apply to the case where all we have are metabarcoding data, with no information of what sequence variants are real and which are not? In such a case, we would have to rely on standard bioinformatic pipelines to infer this and might end up with different conclusions. The question is then whether metabarcoding read numbers will still be useful for predicting species presence or abundance when dealing with metabarcoding-only data?

To address this question, we have added a comparative perspective using a de novo pipeline. In the sections below, we will replicate all key figures and tables as based on the de novo clustering of sequences.

The workflow adopted for the *de novo* analyses is visually summarized in Fig. 1 of the Main Text. In brief, raw read pairs were first demultiplexed using Cutadapt 4.2 (Martin, 2011), with no index mismatches allowed, and a minimum index overlap of nine bases for the ten-base multiplexing indices. Read pairs were then trimmed to remove primer sequences and low-quality ends using Cutadapt 5.0 (Martin 2011). Primer sequences were matched with a maximum 20% error rate, and with required 10 bp overlap. Reads were then trimmed at both ends with a flexible cutoff quality score of 2 (Martin 2011). Reads where either of the primers was not found, which contained ambiguous “N” nucleotides after trimming, or where either read was less than 100 bp long were discarded at this stage. Reads were then further quality filtered using DADA2 1.34.0 (Callahan et al., 2016) to remove read pairs which matched the PhiX genome, or where the number of expected errors exceeded 3 for R1 or 5 for R2. Remaining reads were dereplicated and denoised, independently for R1 and R2, and successfully denoised read pairs were merged, also using DADA2, to produce a set of candidate amplicon sequence variants (ASVs). Candidate ASVs were then filtered through a multi-step process to remove likely sequencing artifacts. Potential cross-contamination was removed using the OptimOTU internal implementation of the UNCROSS2 algorithm (Edgar 2018) with default parameters (expected cross-talk rate 0.05, exponent 1). Potential outlier sequencing errors were removed using the OptimOTU implementation of LULU (Frøslev et al., 2017), with a maximum error rate of 10%, maximum gap length of 1, maximum total gap number of 3, minimum abundance ratio in each sample of 1, and minimum prevalence ratio of 1. Chimeras were detected using the DADA2 consensus-based *de novo* method as well as the reference-based version of UCHIME (Edgar et al., 2011) as implemented in VSEARCH 2.30.0 (Rognes et al., 2016). ASVs identified as chimeric by either method were removed. ASVs were then aligned to the COI hidden markov model (HMM) from Roslin et al. (2022) using Hmmer 3.4 (Eddy 2011). ASVs which did not cover the expected region (positions 245-652 in the HMM) or which had an alignment bit score less than 200 were discarded as being incomplete or off-target. Additionally, ASVs which included stop codons or frame-shifts with respect to the model were discarded as potential sequencing errors or nuclear-mitochondrial pseudogenes (NuMts; Porter and Hajibabaei 2021). ASVs were then taxonomically identified using the version of Protax (Somervuo et al., 2016) described in Roslin et al. (2023), but with an expanded reference database of almost 7.3M sequences across the animal kingdom extracted from BOLD. Finally, aligned ASVs were clustered using the OptimOTU taxonomically constrained clustering algorithm (Furneaux et al., 2025), which produces a full taxonomy at all ranks for all ASVs, using taxonomic identifications from Protax where available and “pseudotaxa” based on single-linkage clustering where Protax was not able to assign taxonomy. Although the OptimOTU algorithm allows clustering thresholds at each rank to vary across the taxonomy, we used static similarity thresholds of 88% identity at phylum rank, 90% at class rank, 93% at order rank, 95% at family rank, 97% at genus rank, and 98% at species rank, based on the default identification thresholds for unmapped reads in mBRAVE. These thresholds are likely higher than real within-taxon variation at the given ranks for many groups. Thus, the pseudotaxa are expected to be conservative, in the sense that sequences in the same pseudotaxon cluster are very likely to actually belong to the same taxon at that rank. At species rank, clustering was performed without taxonomic constraint, so that all operational taxonomic units (OTUs) were delimited *de novo* and according to the same criteria, regardless of whether they belong to identifiable species or not. In order to match the barcoded individuals with the *de novo* OTUs, the individual barcoding sequences were introduced as additional ASVs, which were aligned, taxonomically identified, and clustered along with the ASVs from the metabarcoding.

**References**

Callahan, B. J., McMurdie, P. J., Rosen, M. J., Han, A. W., Johnson, A. J. A., & Holmes, S. P. (2016). DADA2: High-resolution sample inference from Illumina amplicon data. *Nature Methods*, *13*(7), 581–583. <https://doi.org/10.1038/nmeth.3869>

Eddy, S. R. (2011). Accelerated Profile HMM Searches. *PLOS Computational Biology*, *7*(10), e1002195. <https://doi.org/10.1371/journal.pcbi.1002195>

Edgar, R. C., Haas, B. J., Clemente, J. C., Quince, C., & Knight, R. (2011). UCHIME improves sensitivity and speed of chimera detection. *Bioinformatics*, *27*(16), 2194–2200. <https://doi.org/10.1093/bioinformatics/btr381>

Edgar, R. C. (2018). *UNCROSS2: Identification of cross-talk in 16S rRNA OTU tables* (p. 400762). bioRxiv. <https://doi.org/10.1101/400762>

Frøslev, T. G., Kjøller, R., Bruun, H. H., Ejrnæs, R., Brunbjerg, A. K., Pietroni, C., & Hansen, A. J. (2017). Algorithm for post-clustering curation of DNA amplicon data yields reliable biodiversity estimates. *Nature Communications*, *8*(1), Article 1. <https://doi.org/10.1038/s41467-017-01312-x>

Furneaux, B., Anslan, S., Somervuo, P., Hultman, J., Abrego, N., Roslin, T., & Ovaskainen, O. (2025). *OptimOTU: Taxonomically aware OTU clustering with optimized thresholds and a bioinformatics workflow for metabarcoding data* (arXiv:2502.10350). arXiv. <https://doi.org/10.48550/arXiv.2502.10350>

Martin, M. (2011). Cutadapt removes adapter sequences from high-throughput sequencing reads. *EMBnet.Journal*, *17*(1), 10–12. <https://doi.org/10.14806/ej.17.1.200>

Porter, T. M., & Hajibabaei, M. (2021). Profile hidden Markov model sequence analysis can help remove putative pseudogenes from DNA barcoding and metabarcoding datasets. *BMC Bioinformatics*, *22*(1), 256. <https://doi.org/10.1186/s12859-021-04180-x>

R Core Team (2025). *R: A Language and Environment for Statistical Computing*. R Foundation for Statistical Computing, Vienna, Austria. [https://www.R-project.org](https://www.R-project.org/).

Rognes, T., Flouri, T., Nichols, B., Quince, C., & Mahé, F. (2016). VSEARCH: A versatile open source tool for metagenomics. *PeerJ*, *4*, e2584. <https://doi.org/10.7717/peerj.2584>

Roslin, T., Somervuo, P., Pentinsaari, M., Hebert, P. D. N., Agda, J., Ahlroth, P., Anttonen, P., Aspi, J., Blagoev, G., Blanco, S., Chan, D., Clayhills, T., deWaard, J., deWaard, S., Elliot, T., Elo, R., Haapala, S., Helve, E., Ilmonen, J., … Mutanen, M. (2022). A molecular-based identification resource for the arthropods of Finland. *Molecular Ecology Resources*, *22*(2), 803–822. <https://doi.org/10.1111/1755-0998.13510>

Somervuo, P., Koskela, S., Pennanen, J., Henrik Nilsson, R., & Ovaskainen, O. (2016). Unbiased probabilistic taxonomic classification for DNA barcoding. *Bioinformatics*, *32*(19), 2920–2927. <https://doi.org/10.1093/bioinformatics/btw346>

**Table S1. Summary statistics on the overall data, following de novo clustering of metabarcoding sequences.** Shown are the numbers of samples of different type and number of reads passing each step in the bioinformatic pipeline. This Table directly replicates Table 1 of the main paper, but differs in the bioinformatic approach adopted. Thus, the set of steps is also different.

| **Sample set** | **Total** | **Study samples** | **Positive controls** | **Negative controls** |
| --- | --- | --- | --- | --- |
| **n** | 762 × 3 | 45 × 3 | 11 × 3 | 12 × 3 |
| **Raw** | 975 163 733 |  |  |  |
| **Demultiplexed** | 729 910 743 | 47 238 902 | 407 416 | 10 908 |
| **Trimmed** | 727 533 064 | 47 088 602 | 406 070 | 10 153 |
| **Filtered** | 249 512 696 | 16 362 368 | 136 678 | 2 468 |
| **DADA2** | 241 601 921 | 15 991 317 | 135 625 | 1 540 |
| **UNCROSS2** | 241 387 566 | 15 971 427 | 134 457 | 189 |
| **-De novo chimera** | 224 279 586 | 14 938 153 | 134 436 | 156 |
| **-Ref chimera** | 220 761 061 | 14 800 947 | 134 431 | 154 |
| **-Pos control** | 220 506 987 | 14 800 917 | 1 877 | 154 |
| **Aligned** | 220 392 645 | 14 789 537 | 1 877 | 154 |
| **Animal** | 217 919 982 | 14 753 314 | 270 | 154 |
| **Arthropod** | 217 915 769 | 14 753 247 | 270 | 154 |

**Figure S1. Replicability of metabarcoding samples, following *de novo* clustering of metabarcoding sequences.** Replicability is based on samples processed in three replicates and measured through measured by Sørensen (A) and Simpson (B) dissimilarities. Blue points refer to comparisons between replicates of the same sample, red points to comparisons to other samples. This figure directly replicates Fig. 2 of the main text, but differs in the bioinformatic approach adopted.

**Figure S2.** **Comparison between individual barcoding vs. metabarcoding, following *de novo* clustering of metabarcoding sequences.** The upper panels compare OTU richness revealed by individual barcoding (x-axis) to BIN richness revealed by metabarcoding (y-axis), either counting each replicate separately (A), pooling replicates within each sample (B), or including only OTUs present in all three metabarcoding replicates of each sample (C). The line shows a linear model fitted to the data, of which key statistics are inserted in the panels. The lower panels show Sørensen (D) and Simpson (E) dissimilarities between individual barcoding vs. metabarcoding for rows of samples. For the individually sequenced data from each sample, we show dissimilarity with all metabarcoding replicates. Blue points refer to comparisons between observations of the same sample, red points to comparisons to other samples. This figure directly replicates Fig. 3 of the main paper, but differs in the bioinformatic approach adopted.

**Figure S3.** **Dependency of individual barcoding vs. metabarcoding dissimilarity on species richness, following *de novo* clustering of metabarcoding sequences.** The panels show Sørensen dissimilarity (upper panels) and Simpson’s dissimilarity (lower panels) between community composition detected in individually sequenced and metabarcoded samples, plotted as a function of OTU richness in the sample. Since the two methods detected slightly different numbers of OTUs (Fig. S1), we plot the same dissimilarity values against OTU richness resolved by either individual barcoding (left-hand panel) or metabarcoding (right-hand panel). Each data point is an individual sample, with the dissimilarity between the community detected by the two data types shown by the y-axis. This figure directly replicates Fig. 4 of the main paper, but differs in the bioinformatic approach adopted.

**Figure S4.** **The probability of detecting an OTU present in one type of data in the other type of data, following *de novo* clustering of metabarcoding sequences.** Panel (A) shows metabarcode presence as a function of individual abundance, and panel (B) individual presence as a function of the read abundance of this OTU in the metabarcoding data. The lines show a logistic regression model of Y regressed on X. The logistic regression model also includes log(metabarcoding sequencing depth) as a predictor, and the three lines show the prediction for sequencing depth of 500,000 (continuous line), 250,000 (lower dashed line in A, upper dashed line in B), and 1,000,000 (upper dashed line in A, lower dashed line in B) . To resolve individual data points, presence/absence is plotted with jitter around 0 and 1. This figure directly replicates Fig. 5 of the main paper, but differs in the bioinformatic approach adopted.

**Figure S5. The probability of detecting an OTU present in one type of data in the other type of data, following *de novo* clustering of metabarcoding sequences.** A) metabarcode presence as a function of individual abundance, B) individual presence as a function of the read abundance of this OTU in the metabarcoded data. Here, we break down the general pattern shown in Fig S4 by arthropod orders. The lines show logistic regression models of Y regressed on X, including the order-level term of a taxonomic random effect, for sequencing depths 250,000 reads (lower dashed line in A, upper dashed line in B), 500,000 reads (solid line in A and B), and 1,000,000 reads (upper dashed line in A, lower dashed line in B). To resolve individual data points, presence/absence is plotted with jitter around 0 and 1. This figure directly replicates Fig. 6 of the main paper, but differs in the bioinformatic approach adopted.

**Figure S6.** **Abundance of individuals as predicted from metabarcoding reads, following *de novo* clustering of metabarcoding reads.** A) Observed data along with predictions from a hurdle model fitted to log(number of reads) and log(sequencing depth). Predictions are for sequencing depth of 250,000 reads (upper dashed blue line), 500,000 reads (solid blue line) and 1,000,000 reads (lower dashed blue line). B) In-sample predicted vs. actual abundances for a model also including a nested random effect for taxonomic groups at the order, family, and genus levels. The dashed red line is the theoretical 1:1 line for perfect predictions. C) Out-of-sample predicted vs. actual abundances for the same model. This figure directly replicates Fig. 7 of the main paper, but differs in the bioinformatic approach adopted.
